# Trans-activating mutations of the pseudokinase *ERBB3*

**DOI:** 10.1038/s41388-024-03070-9

**Published:** 2024-05-28

**Authors:** Marika K. A. Koivu, Deepankar Chakroborty, Tomi T. Airenne, Mark S. Johnson, Kari J. Kurppa, Klaus Elenius

**Affiliations:** 1 Institute of Biomedicine, and Medicity Research Laboratories, University of Turku, Turku, 20520 Finland; 2 Turku Doctoral Programme of Molecular Medicine, Turku, 20520 Finland; 3 Turku Bioscience Centre, University of Turku and Åbo Akademi University, Turku, 20520 Finland; 4 Structural Bioinformatics Laboratory and InFLAMES Research Flagship Center, Biochemistry, Faculty of Science and Engineering, Åbo Akademi University, 20520 Turku, Finland; 5 Department of Oncology, Turku University Hospital, Turku, 20521 Finland

**Keywords:** Cancer genomics, Mutation, Growth factor signalling

## Abstract

Genetic changes in the *ERBB* family of receptor tyrosine kinases serve as oncogenic driver events and predictive biomarkers for ERBB inhibitor drugs. ERBB3 is a pseudokinase member of the family that, although lacking a fully active kinase domain, is well known for its potent signaling activity as a heterodimeric complex with ERBB2. Previous studies have identified few transforming ERBB3 mutations while the great majority of the hundreds of different somatic *ERBB3* variants observed in different cancer types remain of unknown significance. Here, we describe an unbiased functional genetics screen of the transforming potential of thousands of ERBB3 mutations in parallel. The screen based on a previously described iSCREAM (in vitro screen of activating mutations) platform, and addressing ERBB3 pseudokinase signaling in a context of ERBB3/ERBB2 heterodimers, identified 18 hit mutations. Validation experiments in Ba/F3, NIH 3T3, and MCF10A cell backgrounds demonstrated the presence of both previously known and unknown transforming ERBB3 missense mutations functioning either as single variants or *in cis* as a pairwise combination. Drug sensitivity assays with trastuzumab, pertuzumab and neratinib indicated actionability of the transforming ERBB3 variants.

## Introduction

While tyrosine phosphorylation-mediated signaling is critical for dynamic signaling in multicellular organisms [1], a significant subset of the human protein kinome consist of “kinase-dead” enzymes, with no or very limited enzymatic activity. These pseudokinases have been estimated to comprise 10% of all human protein kinases based on substitutions of conserved amino acid residues with their catalytic sites [2, 3]. While harboring little kinase activity, pseudokinases can still promote signaling and carry out important noncatalytic functions such as serving as scaffolding or docking platforms, regulatory domains, or structural elements [4, 5]. Aberrant signaling by pseudokinases, such as JAK2, ROR1, and TRIB2, has also been demonstrated to be relevant for tumorigenesis in different cancer types [6–10].

The receptor tyrosine kinase (RTK) ERBB3 is another pseudokinase frequently attributed a role in cancer. ERBB3 (HER3) belongs to the ERBB family of RTKs, together with EGFR (ERBB1/HER1), ERBB2 (HER2), and ERBB4 (HER4). *EGFR* and *ERBB2* are well-known human oncogenes and somatic missense mutations, small in-frame indels or amplifications in these genes are clinically used as biomarkers predicting response to EGFR- or ERBB2-targeted therapies [11–13]. While lacking significant kinase activity, ERBB3 can readily heterodimerize with other kinase-competent ERBB receptors and thus promote intracellular signaling. In particular, ERBB3 has been proposed to be the preferred dimerization partner for ERBB2, and to critically contribute to ERBB2-mediated transformation in breast and other cancer types [14–16].

While several monoclonal antibodies targeting ERBB3 have been evaluated in clinical trials, including the antibody-drug conjugate patritumab deruxtecan (clinicaltrials.gov), no ERBB3-targeting therapies are currently clinically available. However, the ERBB2-targeting antibodies trastuzumab and pertuzumab block the formation of signaling-competent ERBB3/ERBB2 heterodimers, and this mechanism has been shown to be critical for the anti-tumor effect [14, 16, 17]. In addition, the ERBB2-targeting tyrosine kinase inhibitors (TKI) such as neratinib and tucatinib may inhibit the signaling through ERBB3/ERBB2 heterodimers [18–22].

While hundreds of different somatic *ERBB3* mutations have been reported in clinical samples representing a number of different cancer types, the functional significance of the variants is mostly unknown. The cBioPortal database [23, 24] currently lists over 800 different somatic *ERBB3* mutations of which the great majority remain without functional annotation. Despite the clear potential of better understanding the predictive value of the *ERBB3* variants for personalizing ERBB-targeting therapies, functional screens to identify gain-of-function alterations in ERBB3 have been hampered by its natural enzymatic inactivity as a pseudokinase.

Here, we describe a functional genetics screen of gain-of-function *ERBB3* variants using a platform based on the previously described iSCREAM [25, 26] pipeline. The approach leveraged expression of 8 055 randomly mutated ERBB3 missense or nonsense variants in Ba/F3 cells and the clonal outgrowth of cell populations expressing the activating variants. As none of the thousands of ERBB3 variants promoted Ba/F3 cell growth when expressed alone – as expected for a pseudokinase – the pipeline was modified with co-expression of ERBB2. The modified iSCREAM and subsequent functional and structural validation experiments resulted in identification of several ERBB3 variants that promoted cellular transformation as heterodimeric complexes with ERBB2. Drug sensitivity analyses confirmed the sensitivity of the activating mutations to ERBB2-targeting therapeutics demonstrating the potential value of the ERBB3 pseudokinase variants as new predictive biomarkers.

## Results

### Somatic *ERBB3* mutations in cancer

*ERBB3* has a somatic mutation frequency of 2.1% in pan-cancer analysis with relatively high frequencies observed *e.g*. in urothelial (7.3–9.8%), endometrial (8.2–12.8%), lung (1.4–8.8%), and colorectal (5.4–6.2%) cancer (cbioportal.org) [23, 24]. Among the 821 observed unique genetic ERBB3 alterations, the cBioPortal database lists only eight missense mutations as oncogenic and 41 variants affecting 11 additional amino acid residues as likely oncogenic (Supplementary Fig. 1).

### Modified cell model for screening of activating variants of the ERBB3 pseudokinase

**Fig. 1:**
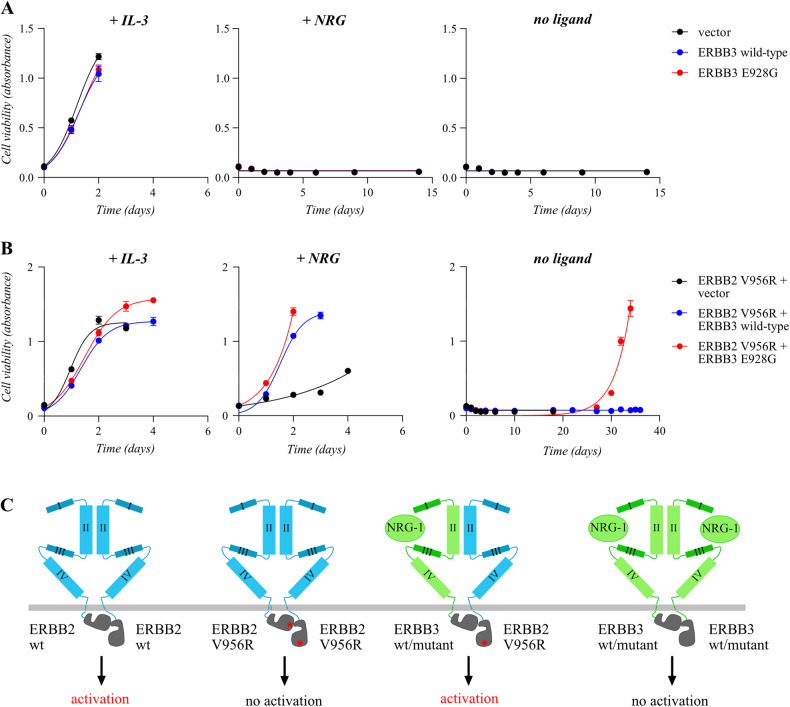
A Ba/F3 cell-based model sensitive to the transactivation potency of ERBB3 variants. **A**, **B** Ba/F3 cells expressing the indicated ERBB3 variants (**A**) or co-expressing the activator-incompetent ERBB2 V956R together with the indicated ERBB3 variants (**B**) were cultured in the presence or absence of IL-3 or the ERBB3 ligand NRG-1. Cell viability was assessed from quadruplicate samples with MTT assay. Mean and SD from an experiment representing one of three independent analyses is shown. **C** Schematic illustration of the constructs used for the analyses. The ERBB2 V956R variant (mutation indicated with a red star) cannot serve as the activator kinase and therefore is not capable of stimulating downstream signaling when present as an ERBB2 V956R homodimer. Wild-type (wt) and mutant ERBB3 variants, however, can serve as the activator kinase for ERBB2 V956R – the potency putatively depending on the nature of the mutation – when stimulated by the NRG-1 ligand. ERBB3 homodimers, in contrast, are all expected to be inactive due to their inherently impaired kinase activity.

Murine lymphoid Ba/F3 cells were selected as the cellular background for screening activating *ERBB3* mutations as we have previously used the model to screen for activating EGFR [25] and ERBB4 [26] variants. The fact that the Ba/F3 cells are critically dependent on exogenous IL-3 for survival and that this dependency can be substituted by ectopic expression of an active RTK [27], allows the use of the cells for a functional readout of RTK-driven growth. However, overexpression of even the well-characterized oncogenic E928G variant of ERBB3 was not sufficient to promote IL-3-independent Ba/F3 cell growth (Fig. 1A), as expected for a pseudokinase in the context devoid of endogenously expressed heterodimeric partners. These observations indicated that Ba/F3 background could not be used to address differential transforming potential of ERBB3 variants in the absence of a kinase-competent partner to heterodimerize with ERBB3.

To modify the model with simultaneous co-expression of ERBB2, but to limit the role of ERBB2 to only serve as the receiver kinase in the ERBB3/ERBB2 dimer, a ERBB2 V956R mutant variant [28–30] was introduced to the Ba/F3 cells (Fig. 1B, C; Supplementary Fig. 2A, B). We had previously demonstrated that co-expression of wild-type ERBB2 together with wild-type ERBB3 in the same model was sufficient to readily promote IL-3-independent growth [31], precluding the use of wild-type ERBB2 as the heterodimer partner. The V956R variant with a valine-to-arginine mutation in the C-lobe of the ERBB2 kinase domain disrupts the capability of the receptor to serve as an activator but does not interfere with its function as a receiver in the transactivation process between two ERBB kinases (Fig. 1C). The “activator-incompetent” V956R was therefore also expected to be incapable of transactivating ERBB2 in a homodimeric ERBB2/ERBB2 complex. Consistent with reduced potential of the ERBB2 V956R mutant in transforming the Ba/F3 cells, it did not promote IL-3-independent growth even in the context of co-expression with wild-type ERBB3 when the complex was not activated by the NRG-1 ligand (Fig. 1B). However, indicating a window for a functional read-out, introducing the oncogenic mutant ERBB3 E928G together with the activator-incompetent ERBB2 V956R did result in IL-3-independent growth (Fig. 1B). The approach also seemed to differentiate between ERBB3 variants with variable transactivation potencies, as the other known oncogenic variants G284R and V104M were clearly less potent as compared to E928G in promoting growth and ERBB3 phosphorylation (Supplementary Fig. 2A, B).

### An expression library of randomly mutated ERBB3 variants

To perform an unbiased screen to study thousands of *ERBB3* mutations in a high-throughput assay, a randomly mutated *ERBB3* cDNA library was created with error-prone PCR, as previously described for the iSCREAM pipeline [25, 26]. As a result, the average mutation frequency in the cDNA library (pDONR221-*ERBB3* library) was estimated to be 1.3 mutations per a 4 029 bp *ERBB3* cDNA insert by Sanger sequencing. The cDNA library was subsequently cloned into a pBABE-gateway retroviral mammalian expression vector. To characterize the distribution of *ERBB3* variants in the library, the *ERBB3* insert from the pBABE-puro-gateway-*ERBB3* library was PCR amplified and deep sequenced using Illumina NovaSeq6000 platform. The analysis indicated that the library was comprised of 9 013 unique *ERBB3* single nucleotide variants out of the 12 088 theoretically possible (74.6%). The resulting amino acid alterations indicated the presence of 8 055 out of the 8 276 theoretically possible amino acid changes (derived by altering a single nucleotide in a codon), resulting in the coverage of 97.3% of all possible *ERBB3* missense or nonsense mutations. Specific missense or nonsense mutation distribution and different transition and transversion mutation distributions are shown in Supplementary Fig. 3.

### Functional genetics screen with the modified iSCREAM pipeline

The *ERBB3* mutation library was transduced into Ba/F3 cells expressing ERBB2 V956R. Cells expressing the mutant expression library or wild-type ERBB3 were cultured in the presence or absence of IL-3, in the presence of 20 ng/ml of the ERBB3 ligand NRG-1 without IL-3, or in the presence of 20 ng/ml of NRG-1 without IL-3 for 48 h prior to complete depletion of both IL-3 and NRG-1 from the culture medium. Pre-incubation for 48 h with NRG-1 was carried out to up-regulate ERBB3 expression and activity in the infected cells (Supplementary Fig. 4) [32]. The cells expressing the *ERBB3* mutation library were able to survive in the complete absence of both IL-3 and NRG-1, while all cells expressing wild-type ERBB3 died (Supplementary Fig. 5). These observations indicated that the *ERBB3* cDNA library included variants that promoted Ba/F3 cell growth by transactivating ERBB2 V956R.

To identify ERBB3 variants enabling Ba/F3 cell transformation, genomic DNA was extracted from the surviving cell populations and the *ERBB3* cDNA inserts were PCR amplified and deep sequenced (>1,300,000×) on Illumina NovaSeq6000 platform. The read counts specific for each *ERBB3* coding sequence variant (normalized to total number of reads at each *ERBB3* locus) in the surviving cell populations were compared to the original transduced IL-3-dependent cell populations, and the enrichment of each specific mutation (fold change) as well as the relative variant frequency of each mutation in the surviving cell pool was estimated (Supplementary Information).

### ERBB3 variants promoting Ba/F3 cell transformation

**Fig. 2:**
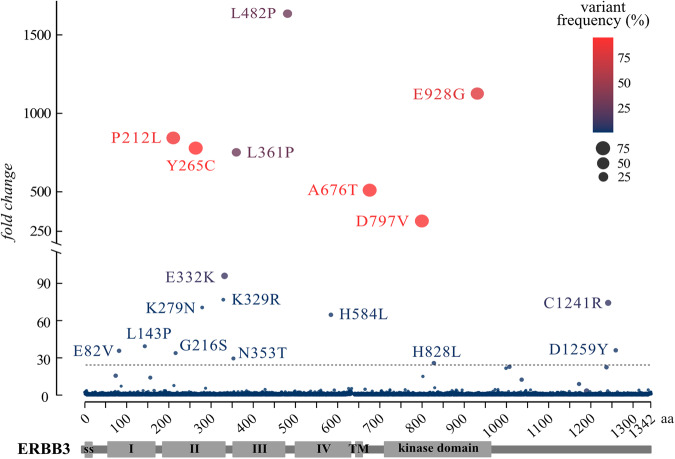
In vitro screen of activating mutations (iSCREAM) of ERBB3. The enrichment of specific ERBB3 mutations in the Ba/F3 cell model is demonstrated as a scatter plot. Ba/F3 cells were transduced with constructs encoding 8 055 different missense or nonsense mutations of ERBB3 together with the activator-incompetent ERBB2 V956R. Observed fold change in the variant frequency of a mutation in the surviving cell pool when compared to the variant frequency of the same mutation in the IL-3-dependent population is shown on the y-axis. The position of the mutated residue on the ERBB3 primary sequence is shown on the x-axis. Variant frequency of the mutation in the final surviving cell pool is depicted with the size of the dot as well as with the intensity of its red color. The dashed horizontal line indicates the fold change level of 25. A pooled analysis of three different experiments is shown. In the independent analyses, the cells were first cultured in the absence of IL-3 with or without 20 ng/ml NRG-1 for two days and subsequently for 15–48 days in the absence of both IL-3 and NRG-1.

The combined results from three independent samples demonstrated that cDNAs encoding 18 ERBB3 missense variants were enriched by at least 25-fold in the Ba/F3 cell populations surviving in the absence of all exogenous ligands (Fig. 2). Seven of the mutations (P212L, Y265C, L361P, L482P, A676T, D797V, and E928G) were enriched by over >100 fold (Fig. 2). The known oncogenic variant E928G was one of the main hits in the screen, validating the approach. In addition, the 18 enriched variants included the missense mutations K329R and E332K annotated as likely oncogenic by cBioPortal (Supplementary Fig. 1).

To validate the hits, 14 individual ERBB3 mutations, each reaching the enrichment level of at least 25 fold, were selected and transduced into Ba/F3 cells either alone or together with ERBB2 V956R. The 14 variants included all seven with enrichment of over 100 fold, as well as seven others from the group of 11 reaching the enrichment level of 25–100-fold.

**Fig. 3:**
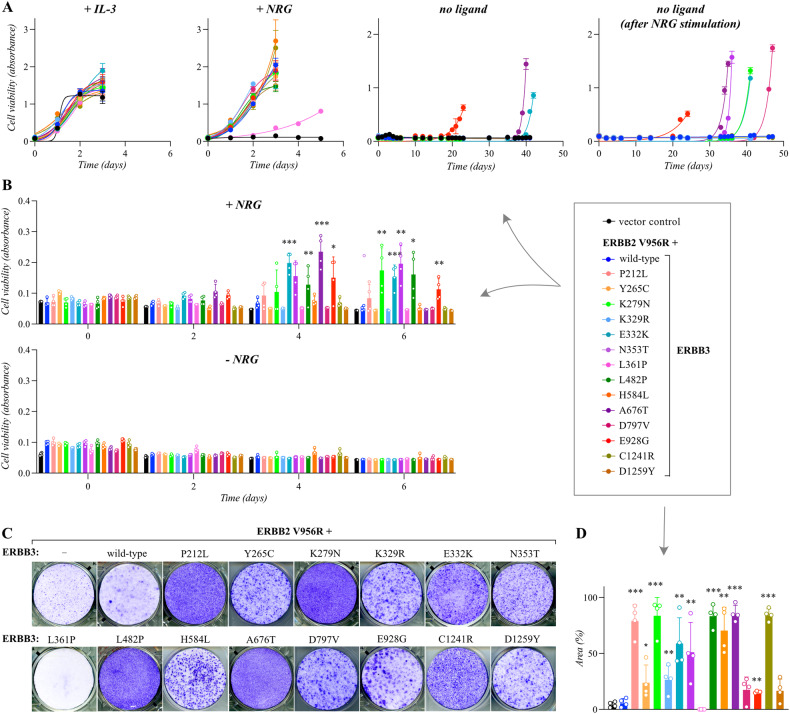
Validation of growth-promoting activity of ERBB3 variants. **A** Ba/F3 cells expressing the indicated ERBB3 variants in the presence of the activator-incompetent ERBB2 V956R were cultured in the presence of IL-3 or 20 ng/ml NRG-1, or in the absence of both (no ligand). The rightmost panel demonstrates an experiment in which the ERBB3 expression was first boosted by NRG-1 stimulation in the absence of IL-3 for two days followed by maintenance in the absence of both ligands. Cell viability was assessed from quadruplicate samples with MTT assay. Mean and SD from an experiment representing one of four independent analyses is shown. **B** MCF-10A cells expressing the same heterodimeric receptor complexes as in A, were cultured in the absence of serum and in the presence or absence of 50 ng/ml NRG-1 for 0, 2, 4, or 6 days. Cell viability was assessed from quadruplicate samples with the MTT assay. Mean and SD from an experiment representing one of three independent analyses is shown. *P < 0.05; **P < 0.01; ***P < 0.001; unpaired two-sample t-test. **C** Focus formation analysis of NIH 3T3 cells expressing the same heterodimeric receptor complexes as in (**A**). The cells were cultured on 6-well plates for 2 weeks and stained with crystal violet. **D** Quantification of the focus formation data, such as shown in (**C**). The area covered by foci was analyzed with ImageJ plugin ColonyArea. Mean and SD from four independent experiments are shown. *P < 0.05; **P < 0.01; ***P < 0.001; unpaired two-sample *t*-test.

When expressed in Ba/F3 cells alone, none of the 14 mutants was able to promote IL-3-independent growth (Supplementary Fig. 6A, B), consistent with the lack of dimerization partner to serve as target for transactivation by ERBB3. However, when co-expressed with the activator-incompetent ERBB2 construct (Supplementary Fig. 6B), expression of E332K, A676T, or E928G induced emergence of IL-3-independent clones (Fig. 3A). When ERBB3 expression was first boosted by a 48-h incubation in the presence of 20 ng/ml of NRG-1 (Supplementary Fig. 4), three additional ERBB3 mutants, K279N, N353T, and D797V, reproducibly also promoted IL-3-independence (Fig. 3A). Importantly, however, in none of the experimental repeats did an IL-3-independent clone emerge from vector- or wild-type ERBB3-transduced clones, regardless of the presence or absence of the heterodimerizing partner ERBB2 V956R.

The L361P mutant seemed to have a slightly aberrant molecular weight in western analyses (Supplementary Fig. 6B), and flow cytometry using an antibody against the extracellular domain of ERBB3 demonstrated lack of the epitope at the cell surface (Supplementary Fig. 6C), consistent with defective maturation and translocation of the variant to the cell surface. All the other ERBB3 mutants were present at the cell surface at similar levels when analyzed by flow cytometry (Supplementary Fig. 7).

### Functional validation of the ERBB3 variants in MCF-10A and NIH 3T3 cells

The 14 ERBB3 mutations selected for further functional characterization were also tested for activity in human MCF-10A mammary epithelial cells and mouse NIH 3T3 fibroblasts. Again, when expressed alone, none of the variants were able to promote survival of serum-starved MCF-10A cells (Supplementary Fig. 8A, B). However, when analyzed in MCF-10A cells co-expressing ERBB2 V956R (Supplementary Fig. 8C), the presence of 50 ng/ml of NRG-1 significantly enhanced the survival and proliferation of cells expressing the ERBB3 variants K279N, E332K, N353T, L482P, A676T, and E928G (Fig. 3B). No significant ligand-independent growth advantage was observed for any of the ERBB3 variants in MCF-10A background (Fig. 3B).

To address the ability of the ERBB3 variants to promote focus formation in NIH 3T3 fibroblasts, the cells infected with retroviruses encoding the different ERBB proteins were seeded in 6-well plates and cultured in 3% serum for two weeks. When expressed alone, the ERBB3 variants K279N, E332K, H584L, and D797V were able to promote formation of foci that covered significantly more area than foci formed by cells expressing wild-type ERBB3 (Supplementary Fig. 9A–C). However, more potent focus formation was observed when ERBB3 variants were analyzed in cells co-expressing ERBB2 V956R (Fig. 3C, D; Supplementary Fig. 9D), and a greater number of ERBB3 variants demonstrated activity. Indeed, 11 out of the 14 hits selected for validation showed transforming activity in the focus formation assay, including P212L, Y265C, K279N, K329R, E332K, N353T, L482P, H584L, A676T, E928G, and C1241R (Fig. 3D).

### ERBB3 mutations demonstrating activity across different cell backgrounds

**Fig. 4:**
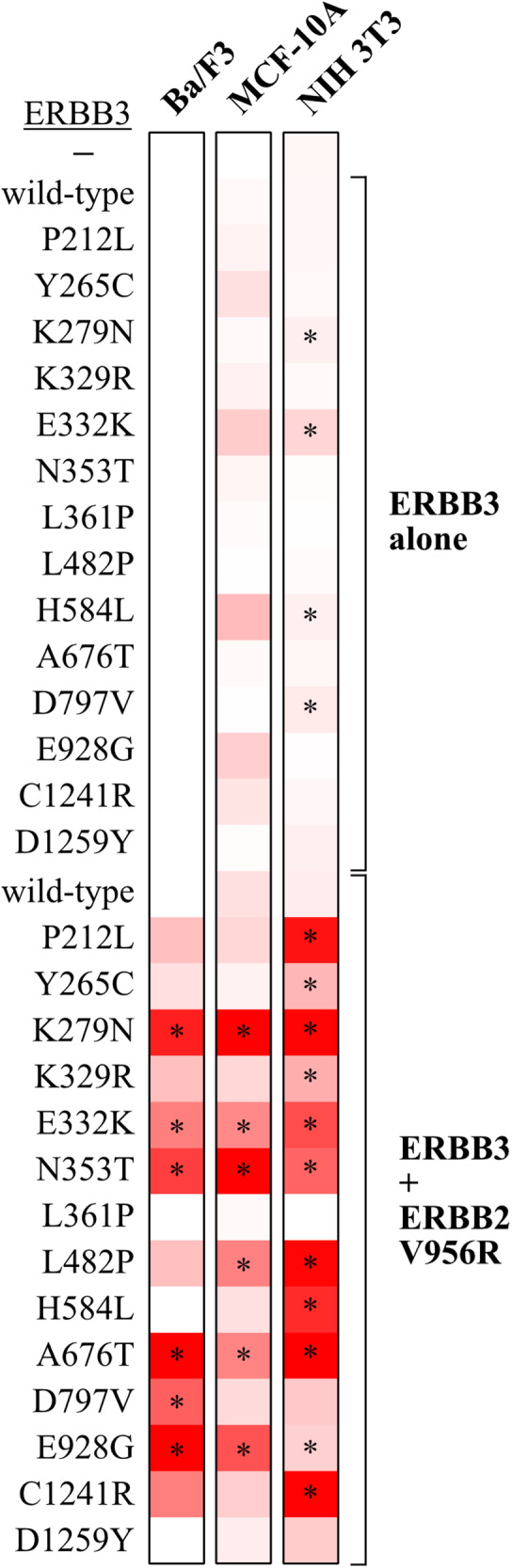
Summary of growth-promoting activity of ERBB3 variants. A heat map presentation of growth promoted by ERBB3 variants when expressed alone (top half) or together with the activator-incompetent ERBB2 V956R (bottom half) in Ba/F3, MCF-10A or NIH 3T3 cells. Ba/F3 cell growth data was normalized from 8 independent MTT experiments in which cells were cultured in the absence of IL-3 after NRG-1 priming, MCF-10A data from 3 independent MTT experiments (+NRG-1), and NIH 3T3 data from 4 independent focus formation experiments. The Ba/F3 data were analyzed as binary – growth or no growth – with white color in the heat map indicating growth in 0/0 experiments and red growth in 8/8 experiments (*P < 0.05; Wilcoxon rank sum test). MCF-10A and NIH 3T3 data are presented as continuous with white color indicating minimal and red maximal growth (*P < 0.05; two-sample *t* test).

Statistical analysis of the growth assays repeated for 3 to 8 times demonstrated that five of the 14 hit variants consistently promoted growth when co-expressed with ERBB2 V956R in all the three cell backgrounds studied (Fig. 4). These were K279N, E332K, N353T, A676T, and E928G. The ERBB3 variant L482P also showed enhanced growth compared to the wild-type cell line in MCF-10A and NIH 3T3 cells but promoted emergence of an IL-3-independent Ba/F3 clone in only one out of eight experiments, not reaching statistical significance. In addition, six variants, P212L, Y265C, K329R, H584L, D797V, and C1241R promoted some activity in two cell backgrounds but reached significance only in one. Only the ERBB3 variants L361P and D1259Y were unable to stimulate growth across all the three cell line models. The mutations were relatively ineffective when expressed alone, with the exception of K279N, E332K, H584L, and D797V that induced statistically more colony formation in NIH 3T3 cells also without concomitant ERBB2 V956R overexpression (Supplementary Fig. 9A, B), suggesting that ERBB3 pseudokinase mutants can harbor gain-of-function properties in cells that, unlike the Ba/F3 cells, express other ERBBs endogenously.

### Long-read sequencing identifies co-occurring mutations

As the mutation frequency in the mutation library was higher than 1 change per *ERBB3* cDNA (i.e. 1.3), the clonal evolution of Ba/F3 cells could favor enrichment of cDNAs that harbor multiple *ERBB3* mutations *in cis*. Relatively weak mutations could also function synergistically to achieve transforming activities. To address whether composite *ERBB3* mutations occurred *in cis* during the clonal evolution in the Ba/F3 screen, the samples analyzed with Illumina NGS – producing reads with the length of approximately 100 bp – were reanalyzed with PacBio High-Fidelity Circular Consensus Sequencing [33] producing reads covering the whole *ERBB3* inserts.

**Fig. 5:**
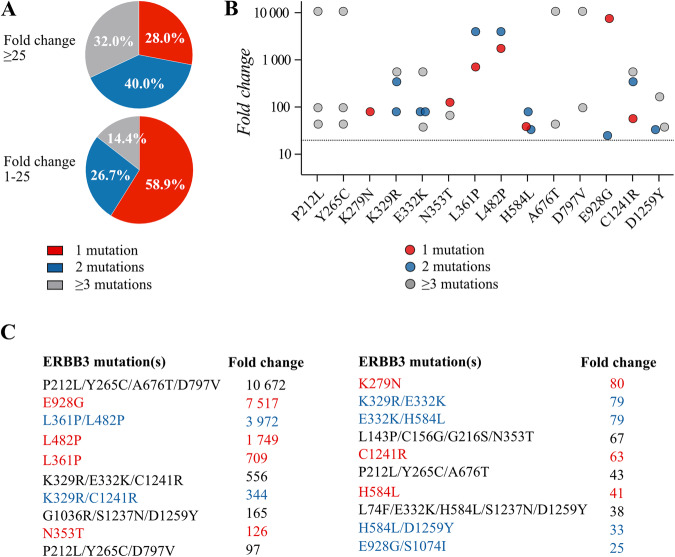
Co-occurring ERBB3 mutations. The samples analyzed with an Illumina NGS platform were re-analyzed with PacBio sequencing producing reads covering the whole ERBB3 inserts. **A** The genotypes of full-length sequencing reads including any one of the 18 ERBB3 mutations that were enriched by ≥25-fold in the iSCREAM by Illumina NGS (Fig. 2) were compared with the full-length reads including any of the 155 mutations that were enriched by values between 1 and 25 and had a variant frequency of >1 in Illumina data. The percentages of mutations occurring either alone, *in cis* with one other ERBB3 mutation, or *in cis* with two or more other ERBB3 mutations is indicated. **B** The 14 ERBB3 mutations that were selected for functional analyses were analyzed for mutations co-occurring *in cis* within the same *ERBB3* reads. Observed maximal fold change enrichment in the IL3-independent population is shown on the y-axis (log10) for mutations occurring alone, *in cis* with one other ERBB3 mutation, or *in cis* with two or more other ERBB3 mutations. The cut-off of fold change value of 25 is depicted with the dotted line. **C** Table listing the co-mutational context and numeric fold change enrichment of the 14 ERBB3 mutations depicted in panel B.

Indeed, when sequencing reads coding for any of the 18 variants enriched by ≥25-fold in the Ba/F3 screen were analyzed, only 28.0% of the full-length reads included only a single ERBB3 mutation (Fig. 5A; Supplementary Information). Instead, 40.0% of the samples included another missense ERBB3 mutation, and 32.0% three or more co-occurring mutations. This distribution of single vs. multiple mutations was significantly different from those ERBB3 mutations that were only enriched by 1–25 fold in the Illumina NGS analysis (*P* = 0.015; Pearson’s Chi-squared test) that more frequently occurred alone (Fig. 5A). Of the 14 variants that were functionally validated, only ERBB3 K279N, N353T, and E928G were predominantly found to be present alone, suggesting that the activity of these mutations did not benefit from additional mutations (Fig. 5B, C). Interestingly, these three mutations composed three out of the five mutations with most consistent functional activity in the validation analyses carried out by expression constructs verified to include only single ERBB3 mutations (Fig. 4). The rest of the mutations co-occurred along with one or several other *ERBB3* mutations *in cis* in the same sequencing reads (Fig. 5B, C).

### Functional significance of mutation co-occurrence

**Fig. 6:**
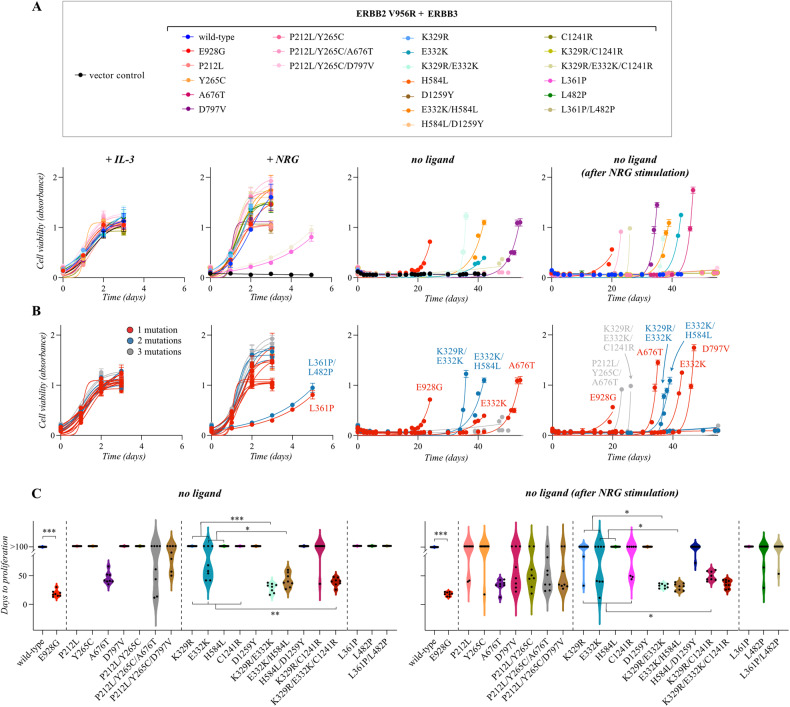
Growth promoted by multiple mutations of ERBB3. **A** Ba/F3 cells expressing the indicated ERBB3 variants in the presence of the activator-incompetent ERBB2 V956R were cultured in the presence of IL-3 or 20 ng/ml NRG-1, or in the absence of both (no ligand). The rightmost panel demonstrates an experiment in which the ERBB3 expression was first boosted by NRG-1 stimulation in the absence of IL-3 for two days followed by maintenance in the absence of both ligands. Cell viability was assessed from quadruplicate samples with MTT assay. Mean and SD from an experiment representing one of four independent analyses is shown. In panel A, the symbols are colored based on the ERBB3 variant, in **B** based on the number of mutations in the ERBB3 insert. **C** Statistical analysis of eight independent analyses carried out as in A. Data are presented as violin plots. Cell clones that did not demonstrate growth within 100 days were all given the value 100 for “Days to proliferation”. *P < 0.05; **P < 0.01; ***P < 0.001; Wilcoxon rank sum test.

To test the hypothesis that some of the ERBB3 variants with weak individual transforming potential functioned synergistically with another alteration to gain activity, the following co-occurring mutations were selected for further analysis: P212L + Y265C + A676T, P212L + Y265C + D797V, P212L + Y265C, L361P + L482P, K329R + E332K + C1241R, K329R + C1241R, K329R + E332K, E332K + H584L, and H584L + D1259Y (Fig. 5B, C). To express the double mutations *in cis* in the same receptor, the mutations were cloned into the same cDNA molecules. The activity of the double or triple mutations in promoting cell survival and growth was then analyzed in Ba/F3 cells as above and compared to the respective single mutations. In Ba/F3 cells, four sets of co-occurring mutations demonstrated significantly enhanced growth as compared to cells expressing the mutants alone (Fig. 6; Supplementary Fig. 10A). These included K329R + E332K, E332K + H584L, K329R + C1241R, and K329R + E332K + C1241R, suggesting that co-mutations complementing K329R or E332K promoted additional activity. However, none of the compound mutations including P212L or Y265C supported growth significantly better than A676T alone, indicating that A676T was not functionally substantiated by additional mutations. Similar to the analyses of single mutants, the activity was restricted to the context of simultaneous overexpression of ERBB2 V956R (Supplementary Fig. 10B). No enhanced growth was observed with cells expressing the double mutant L361P + L482P that, resembling the cells expressing the single mutant ERBB3 L361P, seemed to possess less transforming activity as compared to cells expressing L482P alone (Fig. 6). The L361P + L482P double mutant also phenocopied L361P alone in aberrant migration of the ERBB3 band in western analyses and low or no ligand-stimulated ERBB3 phosphorylation (Supplementary Fig. 6B, Supplementary Fig. 8B, C, Supplementary Fig. 9C-D, Supplementary Fig. 10A), consistent with lack of L361P cell surface expression (Supplementary Fig. 6C).

### Transforming ERBB3 variants facilitate receptor dimerization

**Fig. 7:**
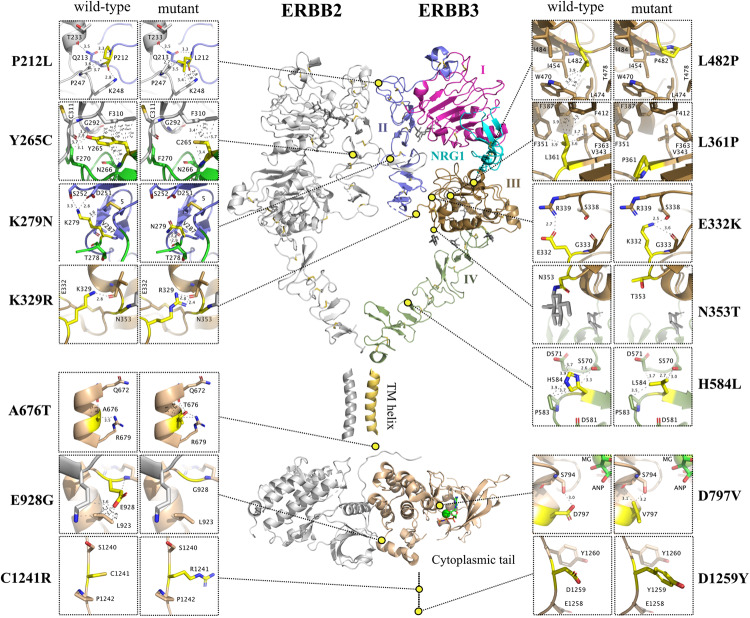
Structural modeling of ERBB3 mutants. The overall composite model was derived using the experimental ERBB2:ERBB3 ectodomain with bound neuregulin-1 (NRG-1) cryoEM structure (PDB:7MN5), TM (transmembrane)-helix NMR structure (ERBB3:ERBB3; PDB:2L9U) and the asymmetric kinase domain X-ray structure (EGFR:ERBB3; PDB:4RIW). For the intrinsically disordered/missing regions (A676T, C1241R and D1259Y), the figure is based on an AlphaFold model (PDB:AF_AFP21860F1). Y265C (zoomed view) was prepared using the dimerization arm stabilizing mutant structure of ERBB2:ERBB3 (PDB:7MN6). ERBB3 domains I-IV are labeled. Residue numbering is based on the full-length sequence of ERBB3 (Uniprot: P21860). The ERBB3 mutations were created using Pymol (The PyMOL Molecular Graphics System, Version 2.4 Schrödinger, LLC.); the rotamers were chosen based on visual inspection.

The 14 ERBB3 mutations identified in our screen were mainly clustered in the extracellular domain and kinase regions (Fig. 7). For example, based on the recent cryo-EM ectodomain structures of ERBB2-ERBB3 [34], K279 is located in domain II of ERBB3 just after the dimerization arm, at the beginning of module 5, a module in direct contact with the dimerization arm of ERBB2, thereby potentially affecting dimer formation and stability; it is known that modules 2–7 of domain II are sensitive to alterations as demonstrated by the oncogenic mutation S310F of ERBB2 that directly stabilizes the dimerization arm of ERBB3 [34]. Between the TM helices and kinase domains, the A676T variant may favor the asymmetric, active conformation of the kinase domain through increasing the stability of the JM region. Improved hydrogen bonding is possible based on *e.g*., the analysis of an Alpha fold model (AF-P21860-F1): an intrasubunit hydrogen bond may form between the side-chain oxygen atom of A676T and both the side-chain nitrogen of R679, conserved in all ERRB receptors, and the main-chain oxygen atom of Q672. The E928G has been previously described as an activating mutation elsewhere [35, 36]. The possible functional consequences of all the missense mutations as analyzed by molecular modeling are listed in the Supplementary Information.

To experimentally address whether the three variants K279N, A676T, and E928G promoted receptor dimerization, NIH 3T3 cells overexpressing the ERBB3 variants alone or together with the ERBB2 V956R variant, were treated for ten minutes with or without 10 ng/ml NRG-1 and subjected to cross-linking with the membrane impermeable compound BS3. Subsequent western analyses demonstrated that all three variants significantly enhanced formation of readily phosphorylated receptor dimers including ERBB3 (Supplementary Fig. 11), consistent with the transforming mutations promoting ERBB3 heterodimerization.

### Sensitivity of mutant ERBB3 transactivation to ERBB inhibitors

**Fig. 8:**
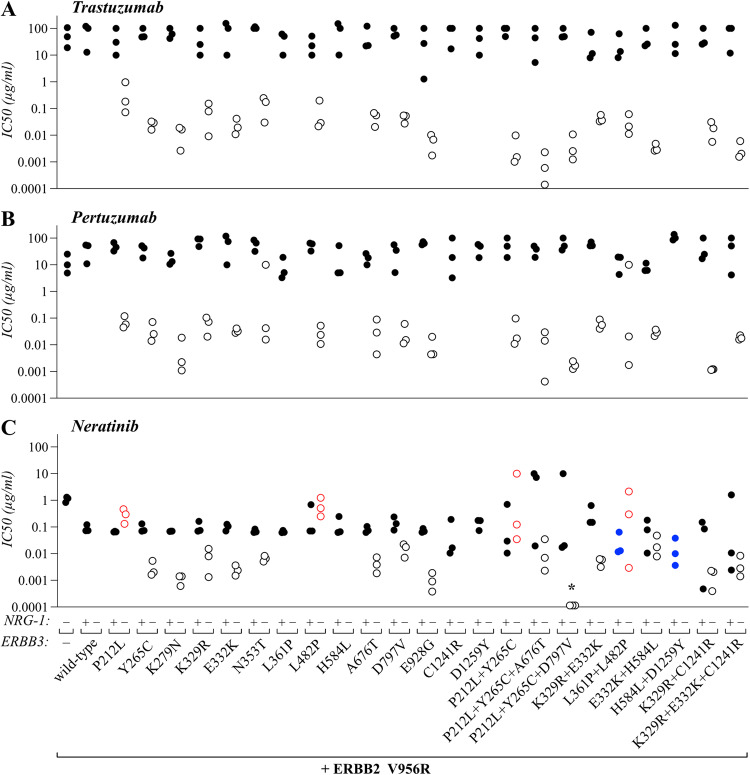
Sensitivity of the ERBB3 variants to ERBB inhibitors. Ba/F3 cells expressing the indicated ERBB3 variants in the presence of the activator-incompetent ERBB2 V956R were cultured in the presence or absence of 20 ng/ml NRG-1 (without IL-3) and treated with trastuzumab (**A**), pertuzumab (**B**), or neratinib (**C**) for 72 h. Cells transduced with an empty vector and cultured in the presence of IL-3 served as a control for off-target toxicity. Cell viability was assessed from triplicate samples with MTT assay. The dots indicate mean IC50 values calculated from three independent experiments. In the absence of NRG-1, all treatments resulted in significantly (*P* < 0.05 with unpaired two-sample t test) reduced IC50’s when compared to cells expressing wild-type ERBB3 and stimulated with NRG-1, with the exceptions indicated by red circles. In the presence of NRG-1, all cell lines were resistant to all drugs (*P* ≥ 0.05 for IC50 compared to wild-type ERBB3 + NRG-1) with the exception indicated with blue dots. No lines surviving the absence of NRG-1 and IL-3 could be established from cell populations expressing wild-type ERBB3, or the L361P, H584L, C1241R, D1259Y or H584L/D1259Y mutation. *P < 0.05 for comparison of IC50 to all single mutations included in the triple mutant variant.

The potential to pharmacologically target the identified ERBB3 variants and to use them as predictive biomarkers for ERBB inhibitors was assessed using the Ba/F3 cell model. As ERBB3 variants alone did not promote ERBB3-dependent growth, and as there are no clinically approved drugs available that would directly block ERBB3, the analyses were carried out in cells co-expressing ERBB2 V956R. In accordance with this approach, the ERBB2/ERBB3 heterodimer has been shown to be an actionable target for ERBB2 antibodies [14, 17, 37, 38]. The cells were cultured for 72 h in the presence of different concentrations of the ERBB2 antibodies trastuzumab or pertuzumab, or the second-generation ERBB tyrosine kinase inhibitor neratinib. The two antibodies did not show an effect for any of the cell lines regardless of the ERBB3 variant expressed when the cells were stimulated with 20 ng/ml NRG-1 for the duration of the experiment (Fig. 8A, B; Supplementary Fig. 12A). This was demonstrated by IC50 values remaining at the >10 µg/ml level, similar to treatment of cells without any ERBB3 expression (vector control) that were cultured in the presence of IL-3, consistent with the well-documented role of NRG-1 in promoting resistance to ERBB inhibitor drugs [19, 39–41]. When the cells were cultured in the absence of NRG-1, significantly lower IC50’s were observed (in the 1–100 ng/ml range) but the sensitivity was similar between all ERBB3 variants (Fig. 8A, B; Supplementary Fig. 12B).

However, when the cells were treated with the TKI neratinib, two of the single ERBB3 mutations, P212L and L482P, as well as the two double mutants P212L + Y265C and L361P + L482P, were resistant even in the absence of NRG-1 (Fig. 8C; Supplementary Fig. 12). The P212L and L482P mutations are located respectively on domains II and III, on either side of the NRG-1 binding site, and hence may result in a receptor conformation mimicking the NRG-1 bound state and leading to resistance to neratinib even in the absence of NRG-1. Cells expressing either of the two double mutations L361P + L482P and H584L + D1259Y were also outliers as they demonstrated moderate, but statistically significant, sensitivity to neratinib also in the presence of NRG-1.

To study the drug responses at the molecular level, phospho-western analyses were also conducted for most of the transforming ERBB3 variants using the Ba/F3 model (Supplementary Fig. 13). As expected, no significant differences were observed between the variants while all displayed suppressed ERBB and AKT phosphorylation in response to the treatments. Taken together, these observations suggest that all the ERBB3 variants can be targeted in ERBB3/ERBB2 heterodimeric complexes with anti-ERBB antibodies or TKI’s but that the sensitivity may depend on the availability of activating ligands in the tumor microenvironment.

## Discussion

Annotation of predictive mutations is critical for evolution of precision oncology, including therapies targeting the ERBB signaling pathway [42–44]. Analyses of the role of ERBB family proteins as predictive biomarkers has focused on EGFR and ERBB2 while there is evidence that individual mutations in *ERBB3* and *ERBB4* could also be biologically relevant [26, 32, 45]. Only a few ERBB3 mutations have previously been characterized for functional significance. These analyses have focused on variants that are prevalent in clinical sample series and have addressed the activity of both extracellular and tyrosine kinase domain mutations of ERRB3 [32, 35, 36, 46, 47].

Here, we addressed the functional significance of 97.3% of all theoretically possible missense and nonsense mutations of ERBB3. To functionally characterize activating mutations in a pseudokinase, a Ba/F3 cell-based model was developed that depended on the activator function of ERBB3’s kinase domain in stimulating transactivation of ERBB2 in an ERBB3/ERBB2 heterodimeric context. This model was used for a modified iSCREAM functional genetics screen to allow unbiased estimation of growth advantage conferred by thousands of random ERBB3 mutations in parallel. While providing proof-of-concept data of transforming potential and actionability of ERBB3 as an oncogene, the current work represents to our knowledge the first attempt to address most possible ERBB3 missense or nonsense mutations in one comprehensive unbiased analysis.

The screen identified 18 ERBB3 variants that were enriched in the Ba/F3 cell population dependent on ERBB3 activator function for survival. Of these, 14 were studied individually in three different cell line models. These validation experiments suggested that 12 out of the 14 variants were more active than wild-type ERBB3 in at least one cell line model and five promoted significant growth advantages in all three cell backgrounds confirming that most of the hits identified in the screen were true positives. Of the 18 variants reaching the cut-off of 25 fold enrichment, three had previously been annotated as oncogenic (E928G) or likely oncogenic (K329R and E332K) by the cBioPortal database. Somatic E928G mutation has been reported in 53 cases with an overrepresentation of breast cancer, and E332K in 29 cases with a high frequency of bladder cancer (Supplementary Fig. 1). K329R has been documented in one case of nasopharyngeal carcinoma and one case of diffuse large B-cell lymphoma. In addition, the variant P212L has been reported as a somatic mutation in a clinical sample representing uterine clear cell carcinoma. These findings, together with the observation that all the ERBB3 variants responded to at least some ERBB inhibitor drugs in the context of ERBB3/ERBB2 complexes, suggest that the modified iSCREAM was able to identify clinically relevant new ERBB3 mutations. However, the majority of the mutations identified in our screen have so far not been reported in patient samples listed in databases.

While the great majority of the identified ERBB3 variants were functionally validated in at least one cell model, only 6 out of 14 consistently promoted growth of Ba/F3 cells when reintroduced to cells from defined expression vectors. However, five additional variants promoted growth in at least once out of eight independent tests, which – albeit not statistically significant – suggested that varying expression levels or other stochastic experimental conditions could have played a role in allowing IL-3-independent growth. Importantly though, the wild-type ERBB3 was never sufficient to promote a growth advantage regardless of the simultaneous ERBB2 co-expression. The fact that three of the ERBB3 mutations did not recapitulate the Ba/F3 cell growth phenotype observed in the initial screen raised the question whether some of the hits from the screen were bystander passenger mutations that accidentally co-occurred in the same cDNA together with the actual driver mutation.

Co-occurrence of mutations in the *ERBB* genes has been observed in clinical samples and can cause enhanced signaling and/or resistance to targeted therapy [21, 48]. For example, *in trans* co-occurring mutations in ERBB3 and ERBB2 were recently reported to promote tumorigenesis and modulate drug sensitivity [21]. Thus, the co-occurrence of ERBB3 mutations *in cis* in the enriched IL-3-independent Ba/F3 populations was addressed by long-read sequencing using PacBio High-Fidelity Circular Consensus Sequencing. The results indicated that, indeed, 72% of the observed hit mutations co-occurred with at least one other ERBB3 missense mutation, and only ERBB3 variants K279N, N353T, and E928G were identified to occur predominantly as single mutations alone. The co-occurrence of multiple mutations in the Ba/F3 cell clones gaining growth advantage was also found to be significantly more frequent as compared to clones displaying little or no enhanced growth. Moreover, functional characterization demonstrated growth-advantage for cells expressing the double or triple ERBB3 mutants K329R + E332K, E332K + H584L, K329R + C1241R, and K329R + E332K + C1241R over cells expressing the same mutations individually. The results support a hypothesis that functionally weaker mutations (such as *e.g*. K329R, E332K, H584L, and C1241R) benefit more from additional mutations, as compared to the stronger drivers that do not seem to obtain similar gain (e.g. E928G). These observations of functional synergism between co-occurring ERBB3 mutations in vitro are also consistent with the findings that ERBB receptors are among the oncogenes that most frequently harbor multiple somatic mutations in clinical cancer samples [48].

Mechanistically, ERBB3 mutations can affect the function of the receptor differently, depending on the location of the mutation and/or the amino acid change [32]. The majority of the ERBB3 variants studied here was located in the extracellular domain but there was also a mutation located in the intracellular juxtamembrane domain and two mutations located in the tyrosine kinase domain (Fig. 7; Supplementary Information). The mutations have the potential to affect hydrogen bonding and hydrophobic interactions, which could result in increased stability of ERBB3 heterodimer or extend the lifetime of the active receptor. K279N might affect the function of the ERBB3/ERBB2 complex due to its close location to the dimerization arm. K329R and E332K have increased hydrogen bonding possibilities, which could translate into added strengths with the K329R + E332K double mutant. N353T would eliminate a N-glycosylation site, which has been shown to have distinct effects [49]. A676T is located at a conserved residue among ERBB receptors [50, 51] and the improved hydrogen bonding possibilities of this variant may favor the asymmetric, active conformation of the kinase domain. Consistent with the concept of the structural changes leading to enhanced ERBB dimerization, all the three variants, K279N, A676T, and E928G, which were functionally tested in western analyses in the presence of a chemical cross-linker, also demonstrated increased ERBB3-ERBB2 dimer formation and activity.

The two hits, L361P and D1259Y, identified in our screen that did not demonstrate any growth advantage in any of the validation models were also found to co-occur *in cis* with other ERBB3 variants in the long-read sequencing. However, validation experiments in which the co-occurring L482P was expressed *in cis* with L361P failed to indicate transforming activity that would have exceeded the activity gained by L482P alone. Flow cytometry analysis demonstrated that ERBB3 L361P was not able to translocate to the cell surface and the variant also exhibited defective ERBB3 phosphorylation and activation of downstream signaling as compared to wild-type ERBB3. We were not able to identify a plausible mechanism, except a role as a passenger of L482P, why this mutation was enriched in our screen. The ERBB3 variant D1259Y when co-expressed *in cis* with H584L, however, promoted Ba/F3 cell growth in one out of eight experiments (Fig. 6C). While not statistically significant, neither of the variants (D1259 nor H584L) never promoted Ba/F3 cell growth alone. These data suggest that the modified iSCREAM method may also produce false positive observations, such as passenger mutations, although at a relatively low frequency.

The screen also failed to identify all previously discovered activating ERBB3 variants, indicating that the screen was not comprehensive for all the 8 055 protein variants included in the cDNA library. We did not *e.g*. identify the most frequently reported ERBB3 variant V104M or the third most frequent G284R (Supplementary Fig. 1), that are both also annotated oncogenic by cBioPortal. However, our control experiments in the Ba/F3 model used for the screen demonstrated that cells expressing V104M were unable to establish an IL-3-independent population and cells expressing G284R were relatively inefficient (Supplementary Fig. 2A). These findings are consistent with the conclusion that the design of our screen to identify mutations promoting transactivation activity by ERBB3 excludes identification of variants conferring oncogenicity by some, not yet determined, mechanisms of action. As was also indicated by our findings that the different ERBB3 variants varied in their transforming potency when expressed in the three different cell backgrounds, it is well-documented that the activity of oncogenes is tissue-dependent [52]. It is also possible in the experimental setup that cells harboring the most potently activating mutation outgrow cells expressing variants with less activity resulting in false negative observations for the latter.

There are currently no approved compounds available in the clinic that directly target ERBB3. Development of ERBB3-targeting TKIs has been hampered by the relatively low intrinsic tyrosine kinase activity of ERBB3. Several therapeutic antibodies have been investigated in clinical trials while positive results from phase 3 trials remain to be documented (reviewed in [10]). Recent work on ERBB3 antibody-drug conjugates has opened up promising new avenues for direct ERBB3 targeting [53]. More indirect targeting of the potently transforming heterodimeric complex of ERBB3 with ERBB2 (with anti-ERBB2 drugs or compounds such as bispecific ERBB3/ERBB2 antibodies) provides another route for activity against ERBB3-driven cancer. Indeed, the mechanism of action of the ERBB2 antibodies pertuzumab and trastuzumab have been shown to involve a blockade in ligand-dependent [17] or -independent [14] ERBB2 dimerization with ERBB3. Consistent with a rationale for indirect targeting of ERBB3 by blocking ERBB2, responses to ERBB2 drugs have been observed in the clinic in patients with ERBB3 mutant cancers [37, 38]. Our analyses with the Ba/F3 cells expressing different ERBB2/ERBB3 variant combinations also suggested that growth and signaling promoted by these heterodimeric complexes is readily suppressed by ERBB2 inhibitors in a number of different ERBB3 mutant contexts. In summary, the screen of activating mutations was able to identify several novel activating mutations with putative predictive value in the pseudokinase ERBB3.

## Materials and methods

### Cell culture

Ba/F3 (DSMZ), NIH 3T3 (ATCC), and Phoenix Ampho cells (a gift from Dr. Garry Nolan) were cultured as previously described [31]. MCF-10A (ATCC) cells were cultured in DMEM/F-12 (Lonza) supplemented with 10% FCS, 50 U/ml penicillin, and 50 U/ml streptomycin, 20 ng/ml EGF (Peprotech), 0.5 mg/ml hydrocortisone (Sigma, #H-0888), 100 ng/ml cholera toxin (Sigma, #C-8052), and 10 µg/ml insulin (Sigma, #I-1882). Cells were routinely tested for *Mycoplasma* infection using MycoAlert (Lonza).

### Expression library of random *ERBB3* mutants

Primer pair 5′-GGGGACAAGTTTGTACAAAAAAGCAGGCTTCACC ATGAGGGCGAACGACGCT-3′ and 5′-GGGGACCACTTTGTACAAGAAAGCTGGGTTTTACGTTCTCTGGGCATTAGCCTT-3′ was used to produce 4093 bp amplicon from pcDNA3.1*ERBB3*-*HA* plasmid [54] containing wild-type *ERBB3* cDNA insert flanked by *aat*B1 and *aat*B2 gateway recombination sites. The amplicon was cloned into the pDONR221 vector [25].

Error-prone PCR was used to generate the expression library encoding randomly mutated human *ERBB3* variants [25]. Primer pair 5′-TTGATGCCTGGCAGTTCCCTA-3′ (binds 78 bp upstream of the attL1 site at the 5’-end of the *ERBB3* insert) and 5′-ATCTTGTGCAATGTAACATCAGAGATT-3′ (binds 80 bp downstream of attL2 site at the 3′-end of the *ERBB3* insert) was used to produce randomly mutated 4 439 bp *ERBB3* cDNA amplicon from pDONR221-*ERBB3*. The PCR product was purified and cloned into a pBABE-puro-gateway vector [25].

### Generation of cell lines with stable ERBB expression

Phoenix Ampho cells were transfected with pBABE-based retroviral expression vectors encoding the ERBB proteins [31]. Retroviral infection with supernatants from Phoenix Ampho cells and selection to produce cell lines with stable ERBB3 expression was carried out, as previously described [25, 31]. Cells with stable expression of activator-incompetent ERBB2 V956R [28–30] were produced by selecting the cells with 500 µg/ml neomycin (Geneticin, Gibco) for 5 days. Selection pressure was subsequently maintained by the presence of 250 µg/ml neomycin in the culture medium. To generate cells with simultaneous expression of both the ERBB2 V956R and ERBB3 library, stable neomycin-selected cells expressing ERBB2 V956R infected with retroviruses produced from pBABE-neo-gateway-*ERBB2* V956R plasmid were transduced with supernatants from Phoenix Ampho cells expressing the pBABE-puro-gateway-*ERBB3* library. When generating cells with simultaneous expression of both ERBB2 V956R and an individual ERBB3 mutant, cells infected with a pBABE-puro-gateway-*ERBB3* plasmid and selected with puromycin were transduced with supernatants from Phoenix Ampho cells expressing pBABE-neo-gateway-*ERBB2* V956R.

### Establishment of IL-3-independent Ba/F3 cell populations and cDNA sample preparation

Ba/F3 cells expressing the ERBB3 mutation library together with ERBB2 V956R were cultured in the presence of IL-3, in the absence of IL-3 but in the presence or absence of 20 ng/ml of the ERBB3 ligand NRG-1 (R&D Systems), or in the absence of both IL-3 and NRG-1 (with or without pretreating the cells first with NRG-1 but without IL-3 for 48 h to promote ERBB expression and signaling). After 15–48 days, the established ligand-independent cell populations were further analyzed.

To amplify *ERBB3* inserts for next-generation sequencing (NGS), genomic DNA was extracted from the Ba/F3 cells (NucleoSpin Tissue, Macherey Nagel) and a total of 24 µg of genomic DNA was used as a template for PCR amplification with the primers: 5′-GGGGACAAGTTTGTACAAAAAAGCAGGCTTCACC ATGAGGGCGAACGACGCT-3′ and 5′-GGGGACCACTTTGTACAAGAAAGCTGGGTTTTACGTTCTCTGGGCATTAGCCTT-3’. The original plasmid containing the cDNA expression library (1 ng) used for transduction was similarly PCR-amplified. A mixture of 1:1 of Phusion (Thermo Fisher Scientific) and Velocity (Bioline) high fidelity DNA polymerases was used for PCR.

### Cell sorting

To quantify ERBB3/ERBB2 expression levels, 500,000 or 3 million Ba/F3 cells were washed three times with PBS and resuspended into 100 µl or 500 µl of flow cytometry staining buffer (FCSB; 3% FBS, 0.09% NaN_3_ in PBS). Non-specific binding was blocked with incubation in 1 µg/1 million cells of IgG (Invitrogen, #10500 C) for 15 min. Human ERBB3/HER3 Alexa Fluor 647-conjugated Antibody (FAB3481R, R&D Systems) and Human ERBB2/HER2 Alexa Fluor 488-conjugated Antibody (FAB1129G, R&D Systems) were used. BD LSRFortessa Cell Analyzer (BD Biosciences) or Sony SH800 Cell Sorter (Sony Biotechnology) were used for cell sorting.

### Analysis of Ba/F3 and MCF-10A cell growth

Ba/F3 cell growth was analyzed using MTT cell viability assays (CellTiter 96 nonradioactive cell proliferation assay; Promega), as previously described [31], in the presence or absence of 20 ng/ml of NRG-1. To analyze MCF-10A cell growth, 2000 cells/well were seeded in quadruplicates into 96-well plate wells, cultured for 0, 2, 4, or 6 days in DMEM/F-12 medium deprived from EGF (Peprotech) and FBS, and supplemented with or without 50 ng/ml NRG-1 and analyzed with the MTT assay. Statistical analysis was performed with the unpaired two-sample t test between cells expressing wild-type or mutant ERBB3 variants. *P* < 0.05 was considered as significant. GraphPad Prism 8 was used for creating the dot plots and bar graphs, and Logistic growth fit was used for the Ba/F3 growth data.

### Western blotting and antibodies

Cells were lysed in lysis buffer and analyzed by western blotting, as previously described [31, 55], using the following primary antibodies: anti-ERBB2 (Thermo Fisher Scientific, Cat#MA-5-14057), anti-ERBB3 (Cell Signaling Technology, Cat#4754), anti-phospho-ERBB2 Tyr1248 (Cell Signaling Technology, Cat#2247), anti-phospho-ERBB3 Tyr1289 (Cell Signaling Technology, Cat#4791), anti-AKT (Cell Signaling Technology, Cat#2920), anti-phospho-AKT (Cell Signaling Technology, Cat#4060), anti-ERK (Cell Signaling Technology, Cat#9102), and anti-phospho-ERK (Cell Signaling Technology, Cat#9101). Loading was controlled using β-actin (Sigma Aldrich, Cat#A5441) antibody. Signals were detected using LI-COR Odyssey CLx and quantified by Image Studio Lite 5.

### Focus formation analyses

Two hundred and fifty thousand NIH 3T3 cells in DMEM supplemented with 3% FBS were seeded into 6-well plates and cultured for 2 weeks. The cells were fixed with 100% methanol for 10 min and stained with 0.4% crystal violet in 25% methanol for 5 min. Wells were washed with milli-Q water five times and the plates were scanned (Epson scanner). Foci were analyzed from four independent experiments using the ImageJ plugin ColonyArea [56]. Statistical analysis was performed by unpaired two-sample t test comparing cells expressing wild-type or mutant ERBB3 variants. *P* < 0.05 was considered as significant. GraphPad Prism 8 was used for creating the dot plots.

### Analysis of ERBB3/ERBB2 dimer formation

To analyze the formation of ERBB3/ERBB2 dimers, stable NIH 3T3 cells expressing the activator-incompetent ERBB2 V956R together with wild-type or mutant ERBB3 were starved overnight and stimulated with 0 or 10 ng/ml NRG-1 for 10 min at 37 °C. After stimulation, the cells were treated with 2 mM BS3 (Thermo Fisher Scientific), as previously described [45]. The formation of ERBB3/ERBB2 dimers was analyzed by western blotting and the signals quantified by Image Studio Lite 5.

### Drug response assays

Ba/F3 cell responses to neratinib (Santa Cruz Biotechnology), pertuzumab (Genentech), or trastuzumab (Genentech) in the absence or presence of 20 ng/ml NRG-1 were analyzed using MTT assays, as previously described [31]. GraphPad Prism 8 was used to present the data as dot plots and the four-parameter inhibitor model was used to calculate IC50 values. For ERBB receptor and downstream signaling analyses, 1 million Ba/F3 cells/ml were incubated in the absence or presence of the drugs and/or 20 ng/ml NRG-1 for five hours. Cells were subsequently lysed and subjected to western blotting. Signals were detected using LI-COR Odyssey CLx and quantified by Image Studio Lite 5. GraphPad Prism 8 was used for creating the bar plots. Statistical analyses for both the MTT and western data were performed using unpaired two-sample t test between cells expressing wild-type or mutant ERBB3 variants. *P* < 0.05 was considered as significant.

### Supplementary information

Supplementary Information

Supplementary Table 2

Supplementary Table 3

## Data Availability

All data generated or analyzed during this study are included in this published article and its Supplementary Information files.

## Code availability

The code for processing the sequencing data is available on the GitHub repository github.com/KE-group/ERBB3_iSCREAM.
