# Supplementary material for: Trans-activating mutations of the pseudokinase ERBB3
Source: Oncogene. 2024 May 28;43(29):2253–65. doi: 10.1038/s41388-024-03070-9 (PMC11245391; doi:10.1038/s41388-024-03070-9)
Supplement: Supplementary file 1 — Supplementary Information [file 41388_2024_3070_MOESM1_ESM.pdf]

Supplementary Information

Supplementary methods

Plasmid constructs for individual mutations

pBABE-neo-gateway vector was prepared by digesting a 1 862 bp fragment encoding the gene for neomycin-resistance (Neo) from pBABE-neo (Addgene, plasmid #1767; http://n2t.net/addgene:1767) with HindIII and NheI (New England Biolabs). The corresponding 737 bp fragment containing the gene for puromycin-resistance was removed from pBABE-puro-gateway (Plasmid #51070; http://n2t.net/addgene:51070) with the same pair of restriction enzymes creating a 6 141 bp fragment of the vector backbone. The insert (1 862 bp) encoding Neo was ligated (T4 DNA ligase; Thermo Scientific) with the digested vector (6 141 bp), thereby incorporating the neomycin resistance gene into the pBABE-gateway retroviral vector. pBABE-neo-gateway-*ERBB2* was subsequently constructed by Gateway cloning [1] (Addgene). pBABE-puro-gateway-*ERBB3* plasmid was constructed by a LR-Gateway recombination reaction between the wild-type pDONR221-*ERBB3* [2] and the pBABE-puro-gateway using LR clonase II mix (Invitrogen). Point mutations were introduced into *ERBB2* and *ERBB3* inserts by site-directed mutagenesis using oligonucleotide primers listed in Supplementary Table 1. All constructs were verified by Sanger sequencing.

Identification of mutations enriched during IL-3-depletion

The purified amplicons were processed with the Nextera DNA Flex Library Preparation Kit (Illumina) to prepare sequencing libraries, which were sequenced on Illumina NovaSeq 6000 with 100-bp paired-end sequencing, producing on average 120 million reads per sample. The NGS data were processed, as previously described [1]. Briefly, trimmed reads [3] were aligned to the "hg19" reference genome (*homo sapiens*) with bwa-mem [4] and variant calling was

performed with bcftools [5,6]. ANNOVAR [7] was used to annotate the variants. The variant frequency of a mutation was defined as the ratio of the number of reads with a particular mutation to the total number of reads aligned to the same locus. The fold change of a mutation between two sampling time points was calculated as a ratio of the final IL-3-independent time point to the initial IL-3-dependent time point. The sequencing data can be found in Supplementary Table 2.

Long-read sequencing

The same PCR amplicons generated from the genomic DNA of surviving cells and the plasmid library (as used for NGS with Illumina short-read sequencing), were reanalyzed with PacBio High Fidelity (HiFi) Circular Consensus Sequencing (CCS) after ligation of adapter sequences and sample barcodes with PCR. Five hundred and fifty ng of PCR product was sent for library preparation and PacBio sequencing to the DNA Sequencing and Genomics Laboratory, Institute of Biotechnology, University of Helsinki. The sequencing library was prepared using SMRTbell Template preparation kit 1.0 and blunt-end adapter. Sequencing of the sample libraries was performed on a PacBio Sequel II instrument using Sequel II Sequencing kit 2.0, Sequel II Binding and Int Ctrl kit 1.0 and Sequel II SMRT Cell 8M following the standard protocol with 0.8 hour pre-extension and 30 hours of collection time (Pacific Biosciences). Demultiplexing and HiFi reads were generated using SMART link software v10 using default settings. CCS reads were generated using the "CCS" tool (https://github.com/PacificBiosciences/ccs) with default settings. HiFi CCS reads were aligned to the "hg38" reference genome (*homo sapiens*) with pbmm2 v1.4.0 (https://github.com/PacificBiosciences/pbmm2) and variant calling was performed with bcftools [5,6]. ANNOVAR [7] was used to annotate the variants. To discern the cDNA molecules harboring composite mutations in the library, samtools [8] (v1.15.1) was used to

query the reads with phred minimum mapping quality (Q) [9] higher than 30 from the sequence alignment in the BAM file. The unique barcodes for the individual reads calling each variant allele were obtained using the 'sam2tsv' script from jvarkit (https://github.com/lindenb/jvarkit). By programmatic investigation of barcodes calling various mutant alleles in the *ERBB3* target gene, we classified two mutations as occurring in *cis* if they were both detected in at least 10 different HiFi CCS long reads (to diminish the false positive detections due to sequencing errors) in at least one sample in the longitudinal sample series (to account for the selective enrichment or depletion of the cDNA harboring composite mutations). The enrichment of "cDNA" haplotypes was calculated as ratio of the allele frequency of the haplotype in final sample to that in the initial sample. The sequencing data can be found in Supplementary Table 3.

Structural analysis of the ERBB3 variants

CryoEM structures with PDB codes 7MN5 and 7MN6 [36], an X-ray structure of EGFR (PDB code 3GOP) and an Alpha fold model (AF-P21860F1) were used for structural analysis. A NMR structure (PDB code 2L9U) was used for visualizing TM helices. The coordinates from two, single particle, high-resolution cryo-electron microscopy structures of the near full-length extracellular domain (ECD) of the heterodimer composed of human ERBB2 and ERBB3 with neuregulin-1β (NRG1β) [10] were obtained from the Protein Data Bank (PDB; [11]: one represents the native ERBB2-ERBB3-NRG1β structure (PDB code 7MN5, 2.93 Å resolution); and the other the oncogenic S310F mutant structure (PDB code 7MN6, 3.09 Å resolution). The modeled regions of both the wild-type and mutant structures are very similar to each other: 7MN6 superposes on 7MN5 with an RMSD value of 0.7 Å (7427 atoms; super command in Pymol; The PyMOL Molecular Graphics System, Version 2.5 Schrödinger, LLC). In the S310F mutant structure, the dimerization arm essential for activation (domain II) of both ERBB2 and

ERBB3 is resolved but in the native structure, the arm is resolved only in the cryo-EM map of ERBB2. The potential effects of point mutations on the structures were made in a conservative way: Bodil [12] and Pymol were used to introduce mutations into the structure, to examine rotamer conformations, to analyze effects, and/or for creating figures.

The coordinates for the protein kinase domain (PKD; EGFR-ERBB3 dimer; PDB code 4RIX [13]) and/or juxtamembrane (JM) segment/region of ERBB2/ERBB3 containing crystal (PDB code 3GOP [14]) and NMR (PDB code 2N2A [15]) structures were obtained from the Protein Data Bank (PDB; [11]). The potential effects of point mutations on the structures were studied in a conservative way: Bodil [12] and Pymol (The PyMOL Molecular Graphics System, Version 2.5 Schrödinger, LLC) were used to introduce mutations into the structure, to examine rotamer conformations, to analyze effects, and/or for creating figures.

Supplementary data

Supplementary Table 1. Primers used for the generation of point mutations into plasmids.

Suppelementary Table 1. Primers used for the generation of point mutations into plasmids.

Gene	Mutation	Forward primer (5'- to -3')	Reverse primer (5'- to -3')
ERBB2	V956R	CATGCGCAAATGTTGGA	ATCATGTAGACATCAATGGTG
ERBB3	P212L	GCTCTTCAGTGTAATGGTCACT	ACAGATGGTCTTGGTCAATGTC
ERBB3	Y265C	GTCTGCAACAAGCTAACTTTCCAG	AAGAGGCTGTGGACAGCG
ERBB3	K279N	CACACCAACTATCAGTATGGAGGAG	GGGATTGGGTTCCAGCTGG
ERBB3	K329R	CCCAGAGCCTGTGAGGGAAC	ACATAGTCCCCCACAAGGCTC
ERBB3	E332K	CCTGTAAGGGAACAGGCTCTG	CTTTGGGACATAGTCCCCCAC
ERBB3	N353T	GATTTGTGACCTGCACCAAGATC	CATCAATGTTGCTCGAGTCCAC
ERBB3	L361P	GGCAACCCGGACTTTCTGAT	CAGGATCTTGGTGCAGTTCACA
ERBB3	L482P	CGACCAGACATCAAGCATAATCGG	CTCTTCCGTAGGCCCCCG
ERBB3	H584L	GCCCCTCTGTGTGAGCAG	CCATCTCGAAAATGGGCACATTG
ERBB3	A676T	AGGACTATGAGGCGATACTTGGAA	TTTATTCTGAATCCGGCGCCC
ERBB3	D797V	CTGGTTCATGTGAGACAACACC	CAGAGAACCCAGAGGCAAATATTG
ERBB3	E928G	GGGGGGCGGTTGGC	CTTCTCTAGCAGGTCTGGTACT
ERBB3	C1241R	CAGAGTCGCCCACTCCACC	TGTGCTGCCCAGAGAGGC
ERBB3	D1259Y	GAATACTATGAATATATGAATCGGC	ATCTGGAGTTGTGCCTG
ERBB3	K329R/E332K	CCCAGAGCCTGTAAGGGAAC	ACATAGTCCCCCACAAGGCTC

Supplementary Table 2. Illumina sequencing data.

Supplementary Table 3. PacBio sequencing data.

Supplementary Table 4. Structural analysis of the ERBB3 variants.

Mutation	Structural analysis
P212L	P212 in β-helical domain II consisting of seven small disulfide-containing modules [16] of ERBB3 is located at the "top"-most position (module II) of the heterodimer interface far away from plasma membrane. This is one of the two key regions that stabilize the globular ectodomain of the ERBB2-ERBB3 heterodimer and, hence, the P212L mutation – a change from a rigid, cyclic nonpolar amino acid to a more flexible one with a large hydrophobic side chain – is poised to contribute essentially to interactions stabilizing the functional receptor.
Y265C	Y265 is located in the dimerization arm of ERBB3 and involved in several intersubunit interactions with its dimerization partner based on the analysis of the ERBB2-ERBB3 dimer structure with a stabilizing oncogenic S310F mutation in ERBB2 (PDB code 7MN6 [10]). The hydrogen bonding of Y265 to G292 and C311 of ERBB2, as well as p-stacking with F310 of ERBB2, seem to be clearly affected by the Y265C mutation. Despite whether similar interactions exist also in the native ERBB2-ERBB3 dimer, it is obvious that the critical position of Y265 in the structure makes it sensitive to any alterations.
K279N	K279 is located in domain II of ERBB3 just after the dimerization arm (β-hairpin loop), at the beginning of the module 5; the module is in direct contact with the dimerization arm of ERBB2. Modules 2-7 of domain II are sensitive to alterations as demonstrated e.g. by the oncogenic mutation S310F of ERBB2 that stabilizes the dimerization arm of ERBB3 (PDB entry 7MN6 [10]). The altered interactions due to the K279N mutation may hence affect the function of the ERBB3/ERBB2 complex.

K329R	K329 is located at the beginning of domain III of ERBB3 and is not in direct contact with ERBB2. The mutation K329R increases the hydrogen bonding possibilities due to the length of the side chain and the guanidinium group: K329R has the potential to form an additional hydrogen bond to the main-chain oxygen atom of V352 in comparison to K329 and has hence potential to strengthen the local structure.
E332K	E332, adjacent to a disulphide bridge, is located near K329 but points in the opposite direction, towards domain II of ERBB3. In the native structure, E332 forms a salt bridge with R339; the E332K mutant has potential to form strong hydrogen bonds with S338 and G333.
N353T	The N353 is one of the ten N-glycosylation sites in ERBB3 [17]. The N353T mutation eliminates site-specific N-glycosylation, which was demonstrated with Western blot analysis of cells expressing the N353T mutant. As there is evidence that site-specific glycosylation can have distinct effects [18], the N353T mutation is likely to affect ERBB3 function.
L361P	L361 is deeply buried residue in a hydrophobic environment and located inside the barrel-shaped β-helix domain III. The L361P mutation would alter the core architecture of domain III, a domain playing a central role in formation of the active heterodimeric receptor and binding the growth factor ligand.
L482P	L482 is in a hydrophobic environment located at the end of the barrel-shaped β-helix domain III situated between the neuregulin-1 binding site and domain IV. L482P mutation is likely to affect the local conformation at the end of domain III.
H584L	H584 is located at the membrane-proximal domain IV. H584 of ERBB3 and equivalent residues of EGFR (H590) and ERBB4 (N588) are thought to be important contact residues with domain II in a tethered (inactive) conformation [16]. In ERBB2 the equivalent residue is a hydrophobic F614 and phenylalanine at this position is thought to cause inability of ERBB2 to form the closed, tethered form. The hydrophobic leucine of the H584L variant, in addition to altering interactions withing the domain IV in comparison to the wild-type structure, may affect the ability of ERBB3 to form the closed conformation and thereby support activation of the receptor.
A676T	The A676T variant may favor the asymmetric, active conformation of the kinase domain through increasing the stability of the JM region; improved hydrogen bonding is possible based on *e.g.* the analysis of an Alpha fold model (AF-P21860-F1). For example, an intrasubunit hydrogen bond may form between the side-chain oxygen atom of A676T and both the side-chain nitrogen of R679 and main-chain oxygen atom of Q672. The arginine residue at the position equivalent to R679 of ERBB3 is conserved in all ERRB receptors. Position A676 is equivalent to T678 of EGFR [14] and T686 [19] of ERBB2, both of which are known to be phosphorylated. Phosphorylation at this position is suggested to have substantial effects [20], including decreased affinity for EGF and loss of EGF-stimulated receptor autophosphorylation [21–24]. The Alpha fold model (AF-P21860F1) was used to create the figure.
D797V	The D797V mutant – a change from hydrophilic to hydrophobic residue – is located at the entrance to the ATP-binding site and may affect ligand entry and/or local stability of the ligand-binding site.
E928G	E928G has been previously described elsewhere [13,25].
C1241R	C1241R is located at the C-terminal tail in intrinsically disordered region. It is not clear how this mutation would affect the receptor. The Alpha fold model (AF-P21860F1) was used to create the figure.
D1259Y	The D1259Y mutant is located at the C-terminal tail, adjacent to another tyrosine residue. This mutant has the potential to be phosphorylated or affect the phosphorylation of the adjacent, or other, tyrosine residues along the tail. No experimental 3D structural data are available for the C-terminal tail, which is intrinsically disordered. The Alpha fold model (AF-P21860F1) was used to create the figure.
K329R/E332K	Moreover, the added strength of the K329R-E332K double mutant would further support the structural changes and explain why K329R exerts an effect in concert with the E332K mutation but not on its own.

Additional resources

The Illumina and PacBio raw and processed sequencing data are available for academic purposes by request.

References

1. Chakroborty D, Kurppa KJ, Paatero I, Ojala VK, Koivu M, Tamirat MZ, et al. An unbiased in vitro screen for activating epidermal growth factor receptor mutations. J Biol Chem. 2019;294:9377–89.

2. Koivu MKA, Chakroborty D, Tamirat MZ, Johnson MS, Kurppa KJ, Elenius K. Identification of Predictive ERBB Mutations by Leveraging Publicly Available Cell Line Databases. Mol Cancer Ther. 2021;20:564–76.

3. Bolger AM, Lohse M, Usadel B. Trimmomatic: A flexible trimmer for Illumina sequence data. Bioinformatics. 2014;30:2114–20.

4. Li H. Aligning sequence reads, clone sequences and assembly contigs with BWA-MEM. arXiv: Genomics. 2013.

5. Li H, Barrett J. A statistical framework for SNP calling, mutation discovery, association mapping and population genetical parameter estimation from sequencing data. Bioinformatics. 2011;27:2987–93.

6. Danecek P, Bonfield JK, Liddle J, Marshall J, Ohan V, Pollard MO, et al. Twelve years of SAMtools and BCFtools. GigaScience. 2021;10:1–4.

7. Wang K, Li M, Hakonarson H. ANNOVAR: functional annotation of genetic variants from high-throughput sequencing data. Nucleic Acids Research. 2010;38:e164–e164.

8. Li H, Handsaker B, Wysoker A, Fennell T, Ruan J, Homer N, et al. The Sequence Alignment/Map format and SAMtools. Bioinformatics. 2009;25:2078–9.

9. Ewing B, Green P. Base-calling of automated sequencer traces using phred. II. Error probabilities. Genome Research. 1998;8:186–94.

10. Diwanji D, Trenker R, Thaker TM, Wang F, Agard DA, Verba KA, et al. Structures of the HER2-HER3-NRG1β complex reveal a dynamic dimer interface. Nature. 2021;600:339–43.

11. Berman HM, Westbrook J, Feng Z, Gilliland G, Bhat TN, Weissig H, et al. The Protein Data Bank. Nucleic Acids Research. 2000;28:235–42.

12. Lehtonen J V., Still DJ, Rantanen V V., Ekholm J, Björklund D, Iftikhar Z, et al. BODIL: a molecular modeling environment for structure-function analysis and drug design. Journal of Computer - Aided Molecular Design. 2004;18:401–19.

13. Littlefield P, Liu L, Mysore V, Shan Y, Shaw DE, Jura N. Structural analysis of the EGFR/HER3 heterodimer reveals the molecular basis for activating HER3 mutations. Sci Signal. 2014;7:ra114.

14. Red Brewer M, Choi SH, Alvarado D, Moravcevic K, Pozzi A, Lemmon MA, et al. The Juxtamembrane Region of the EGF Receptor Functions as an Activation Domain. Mol Cell. 2009;34:641–51.

15. Bragin PE, Mineev KS, Bocharova O V., Volynsky PE, Bocharov E V., Arseniev AS. HER2 Transmembrane Domain Dimerization Coupled with Self-Association of Membrane-Embedded Cytoplasmic Juxtamembrane Regions. Journal of Molecular Biology. 2016;428:52–61.

16. Cho HS, Leahy DJ. Structure of the extracellular region of HER3 reveals an interdomain tether. Science. 2002;297:1330–3.

17. Black LE, Longo JF, Carroll SL. Mechanisms of Receptor Tyrosine-Protein Kinase ErbB-3 (ERBB3) Action in Human Neoplasia. American Journal of Pathology. 2019;189:1898–912.

18. Yokoe S, Takahashi M, Asahi M, Seung HL, Li W, Osumi D, et al. The Asn418-linked N-glycan of ErbB3 plays a crucial role in preventing spontaneous heterodimerization and tumor promotion. Cancer Res. 2007;67:1935–42.

19. Guy PM, Platko J V., Cantley LC, Cerione RA, Carraway KL. Insect cell-expressed p180erbB3 possesses an impaired tyrosine kinase activity. Proceedings of the National Academy of Sciences. 1994;91:8132–6.

20. Thiel KW, Carpenter G. Epidermal growth factor receptor juxtamembrane region regulates allosteric tyrosine kinase activation. Proceedings of the National Academy of Sciences of the United States of America. 2007;104:19238–43.

21. Adak S, Yang KS, Macdonald-Obermann J, Pike LJ. The membrane-proximal intracellular domain of the epidermal growth factor receptor underlies negative cooperativity in ligand binding. The Journal of biological chemistry. 2011;286:45146–55.

22. Magun BE, Matrisian LM, Bowden GT. Epidermal growth factor. Ability of tumor promoter to alter its degradation, receptor affinity and receptor number. Journal of Biological Chemistry. 1980;255:6373–81.

23. Hunter T, Ling N, Cooper JA. Protein kinase C phosphorylation of the EGF receptor at a threonine residue close to the cytoplasmic face of the plasma membrane. Nature. 1984;311:480–3.

24. Shoyab M, De Larco JE, Todaro GJ. Biologically active phorbol esters specifically alter affinity of epidermal growth factor membrane receptors. Nature. 1979;279:387–91.

25. Collier TS, Diraviyam K, Monsey J, Shen W, Sept D, Bose R. Carboxyl group footprinting mass spectrometry and molecular dynamics identify key interactions in the HER2-HER3 receptor tyrosine kinase interface. J Biol Chem. 2013;288:25254–64.

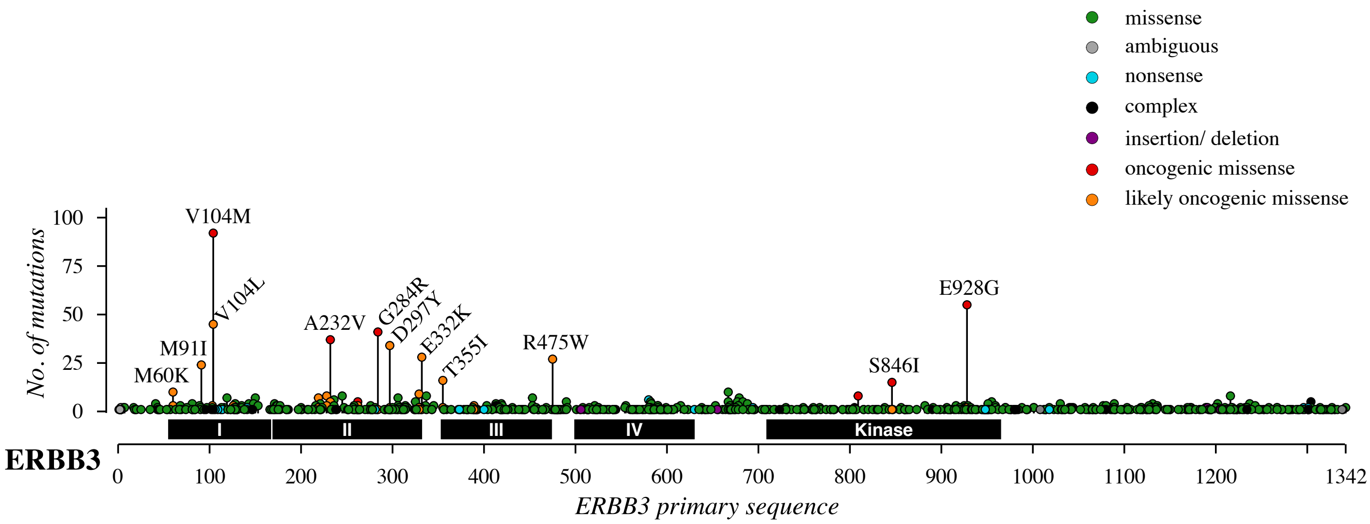

Supplementary Figure 1. ERBB3 mutations in cancer.
ERBB3 coding sequence mutations reported in the cBioPortal database (v5.1.6) are shown. Total number of mutations = 1 605, number of unique mutations = 796. Height of the lollipop (y-axis) indicates the number of mutations observed at the amino acid position along the ERBB3 primary sequence (x-axis). Mutations annotated "oncogenic" or "likely oncogenic" by cBioPortal and listed at least 10 times in the database are indicated by text.

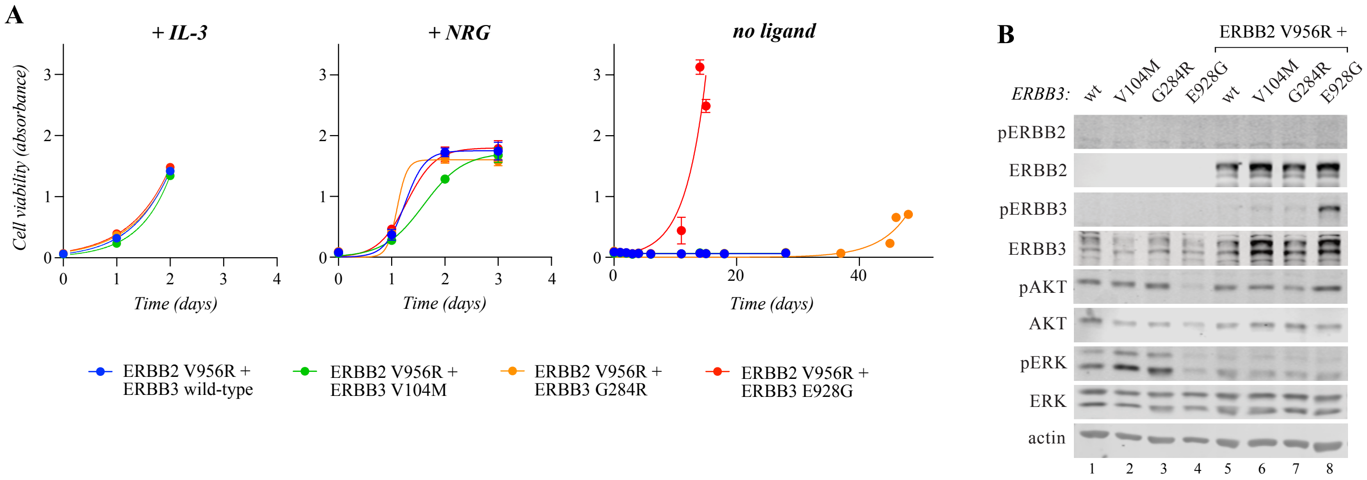

Supplementary Figure 2. Establishing the Ba/F3 cell-based model sensitive to the transactivation potency of ERBB3 variants.

A: Ba/F3 cells expressing the indicated ERBB3 variants in the presence of the activator-incompetent ERBB2 V956R were cultured in the presence of IL-3 or 20 ng/ml NRG-1, or in the absence of both (no ligand). Cell viability was assessed from quadruplicate samples with MTT assay. Mean and SD from an experiment representing one of three independent analyses is shown.

B: Western analysis of Ba/F3 cells expressing the indicated ERBB variants cultured in the presence of IL-3. wt, wild-type.

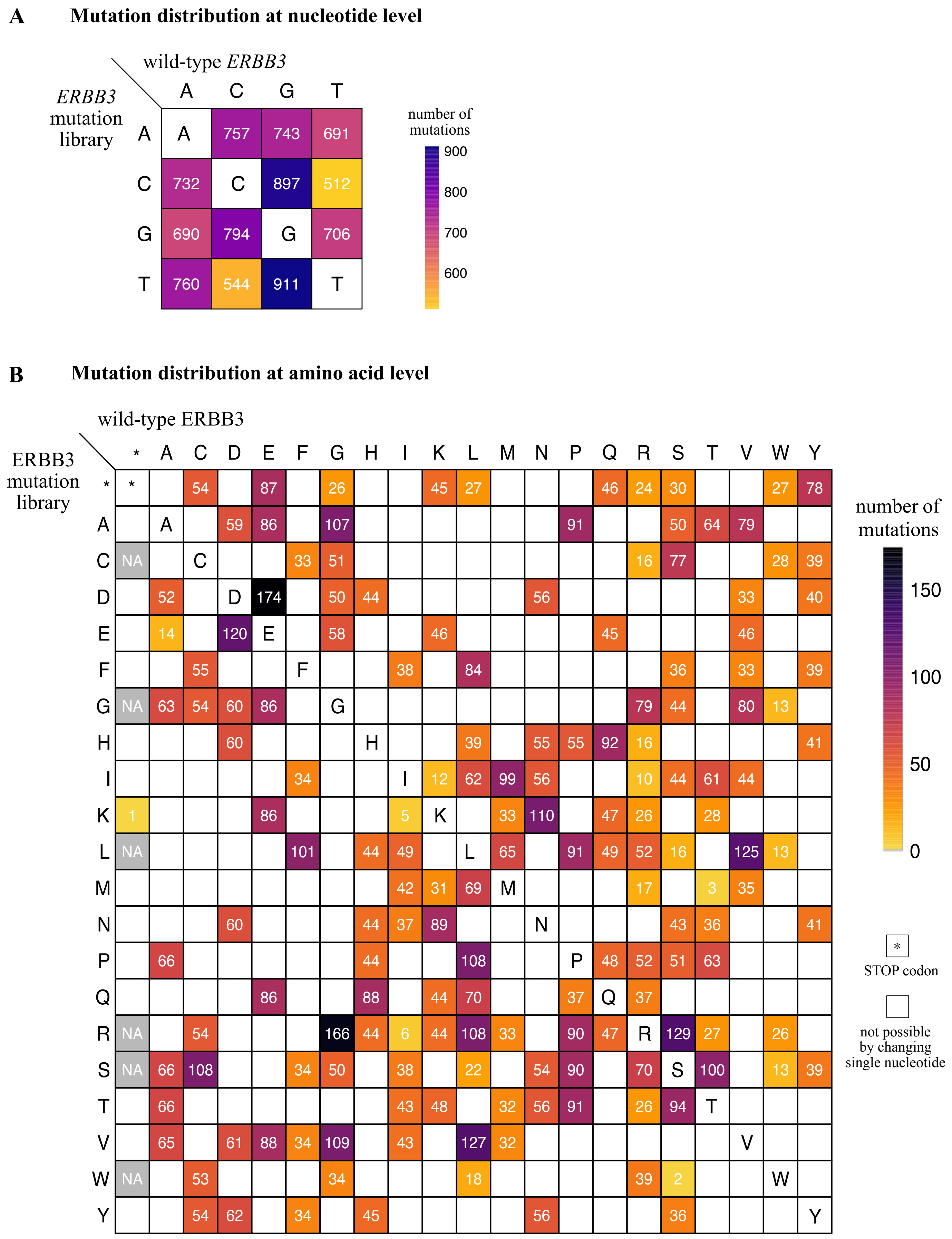

Supplementary Figure 3. Distribution of ERBB3 mutations in the expression library
Distribution of the observed nucleotide changes (A) and amino acid changes (B) in the *ERBB3* mutation library. The cells at the intersections of the labels on the columns and the rows in both matrices show the number of mutations with that specific change (either nucleotide or amino acid).

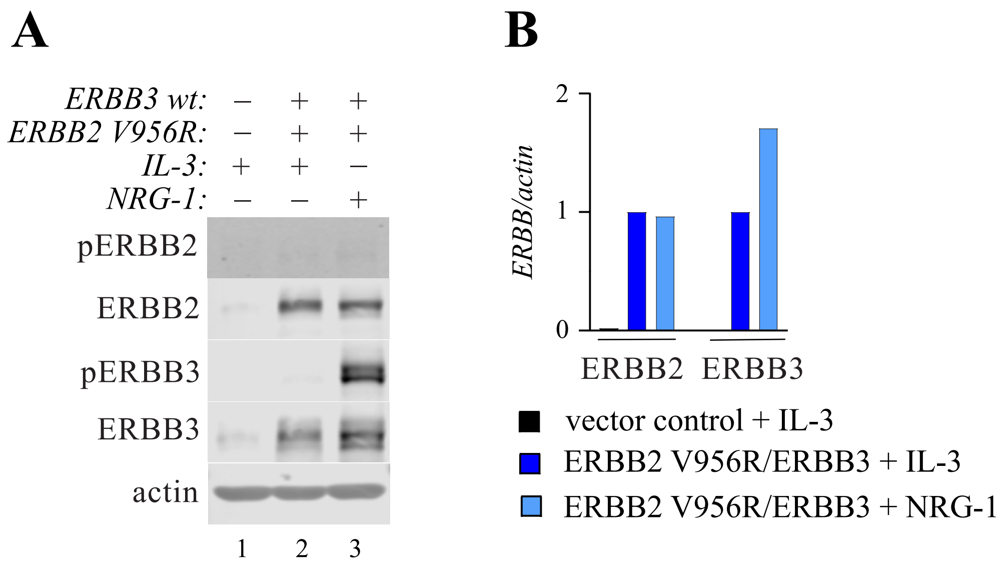

Supplementary Figure 4. Effect of NRG-1 stimulation on ERBB3 expression and phosphorylation.
A: Ba/F3 cells expressing wild-type ERBB3 together with the activator-incompetent ERBB2 V956R were cultured for 48 hours in the presence of IL-3 or 20 ng/ml NRG-1. Cells transduced with an empty vector and cultured in the presence of IL-3 served as a control (lane 1). Cells were lysed and subjected to western analysis with the indicated antibodies. wt, wild-type.
B: Densitometric quantification of total ERBB protein expression in western analysis shown in panel A.

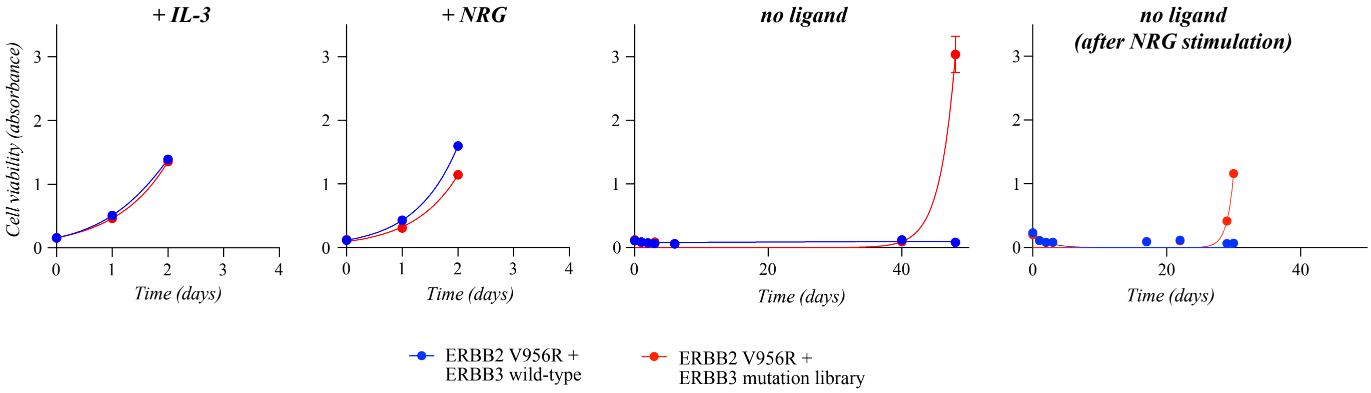

Supplementary Figure 5. Ligand-independent Ba/F3 cell growth promoted by ERBB3 mutation library. MTT growth analysis of Ba/F3 cells expressing ERBB2 V956R together with either wild-type ERBB3 or the expression library of randomly mutated ERBB3 variants. Cells were cultured in the presence of IL-3 or 20 ng/ml NRG-1, or in the absence of both (no ligand). The rightmost panel demonstrates an experiment in which the ERBB3 expression was first boosted by NRG-1 stimulation in the absence of IL-3 for two days followed by maintenance in the absence of both ligands. Mean and SD are shown (n = 4).

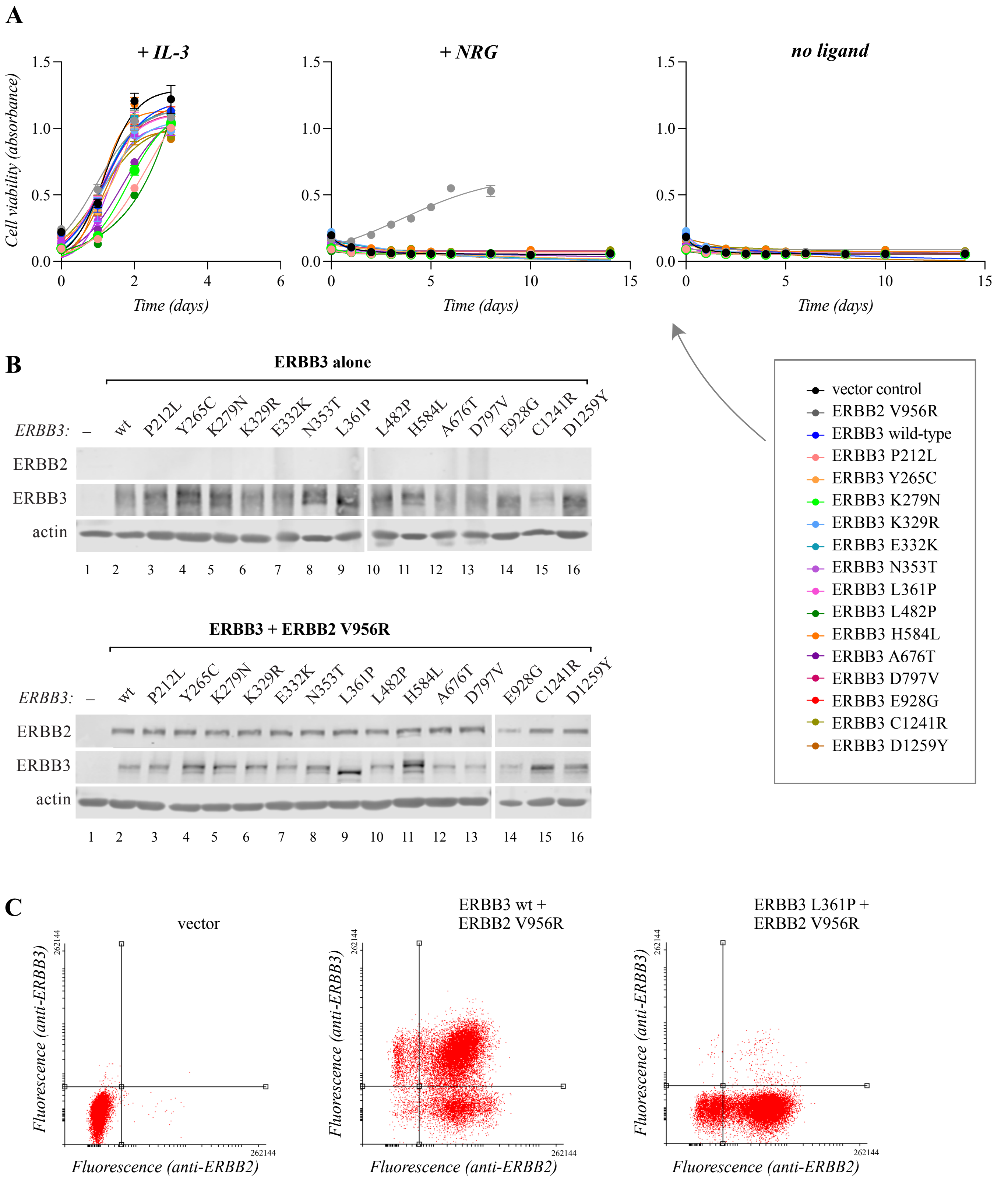

Supplementary Figure 6. ERBB3 variants in Ba/F3 cells.

A: Ba/F3 cells expressing the indicated ERBB3 variants (without ERBB2 V956R co-expression) or ERBB2 V956R were cultured in the presence of IL-3 or 20 ng/ml NRG-1, or in the absence of both (no ligand). Cell viability was assessed from quadruplicate samples with MTT assay. Mean and SD from an experiment representing one of four independent analyses is shown.

B: Western analysis of Ba/F3 cells expressing the indicated ERBB3 variants in the absence or presence of the activator-incompetent ERBB2 V956R. Cells were cultured in the presence of IL-3.

C: Flow cytometry analysis of cell surface expression of ERBB3 and ERBB2 in Ba/F3 cells transduced with vector alone (left) or expressing both wild-type ERBB3 and ERBB2 V956R (center), or both ERBB3 L361P and ERBB2 V956R (right). Alexa Fluor 488-conjugated anti-ERBB2 and Alexa Fluor 647-conjugated anti-ERBB3 were used for the analysis. Cells were cultured in the presence of IL-3.

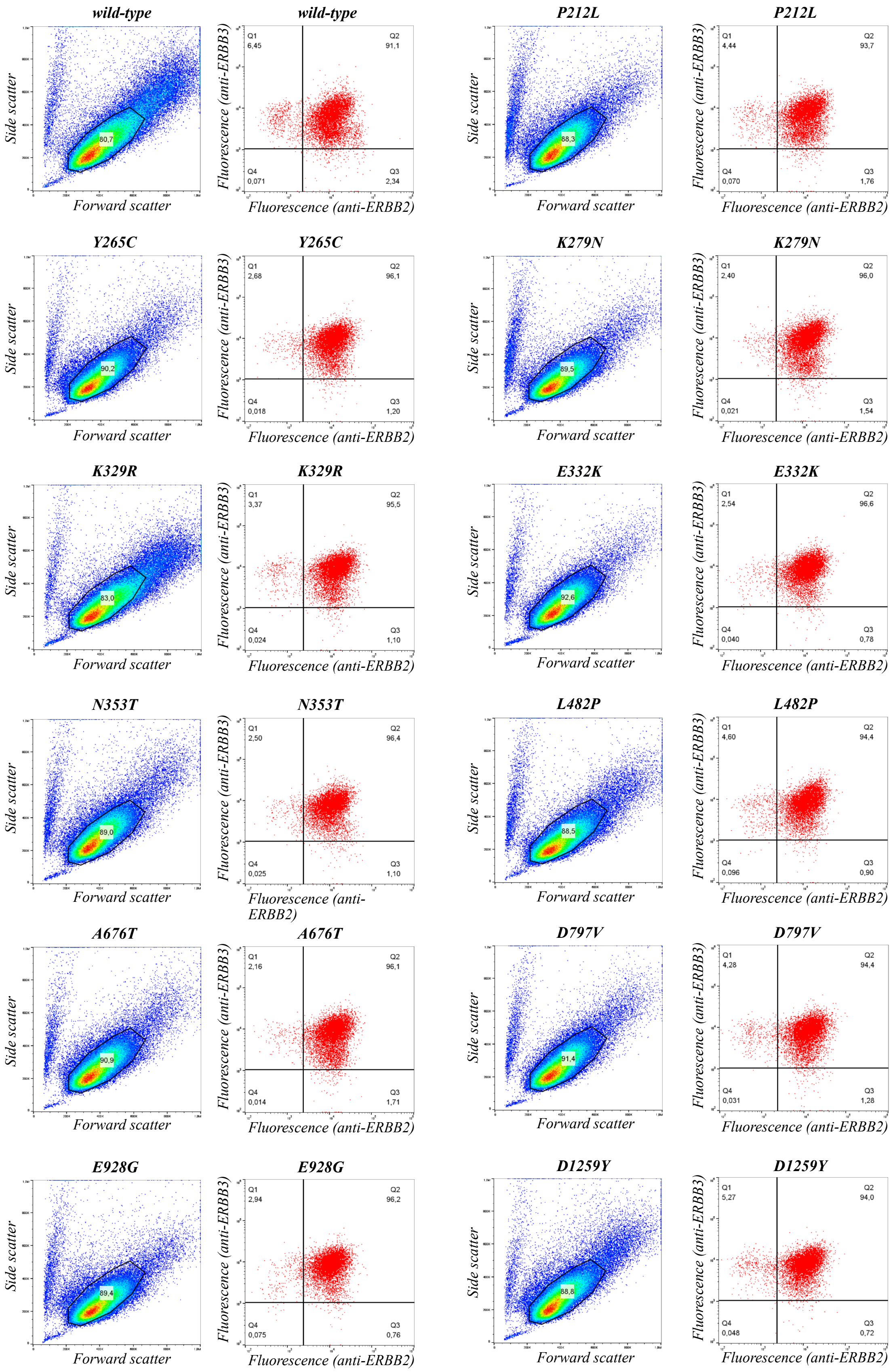

Supplementary Figure 7. Flow cytometry analysis of Ba/F3 cells expressing ERBB3 variants.
Ba/F3 cells expressing the indicated ERBB3 variants together with the activator-incompetent ERBB2 V956R were analyzed for cell surface ERBB2 and ERBB3 expression by flow cytometry. Alexa Fluor 488-conjugated anti-ERBB2 and Alexa Fluor 647-conjugated anti-ERBB3 were used. Cells were maintained in the presence of IL-3. FlowJo software (v10.9) was used for creation of images. The analysis demonstrated similar cell surface expression for both receptors in all the cell lines.

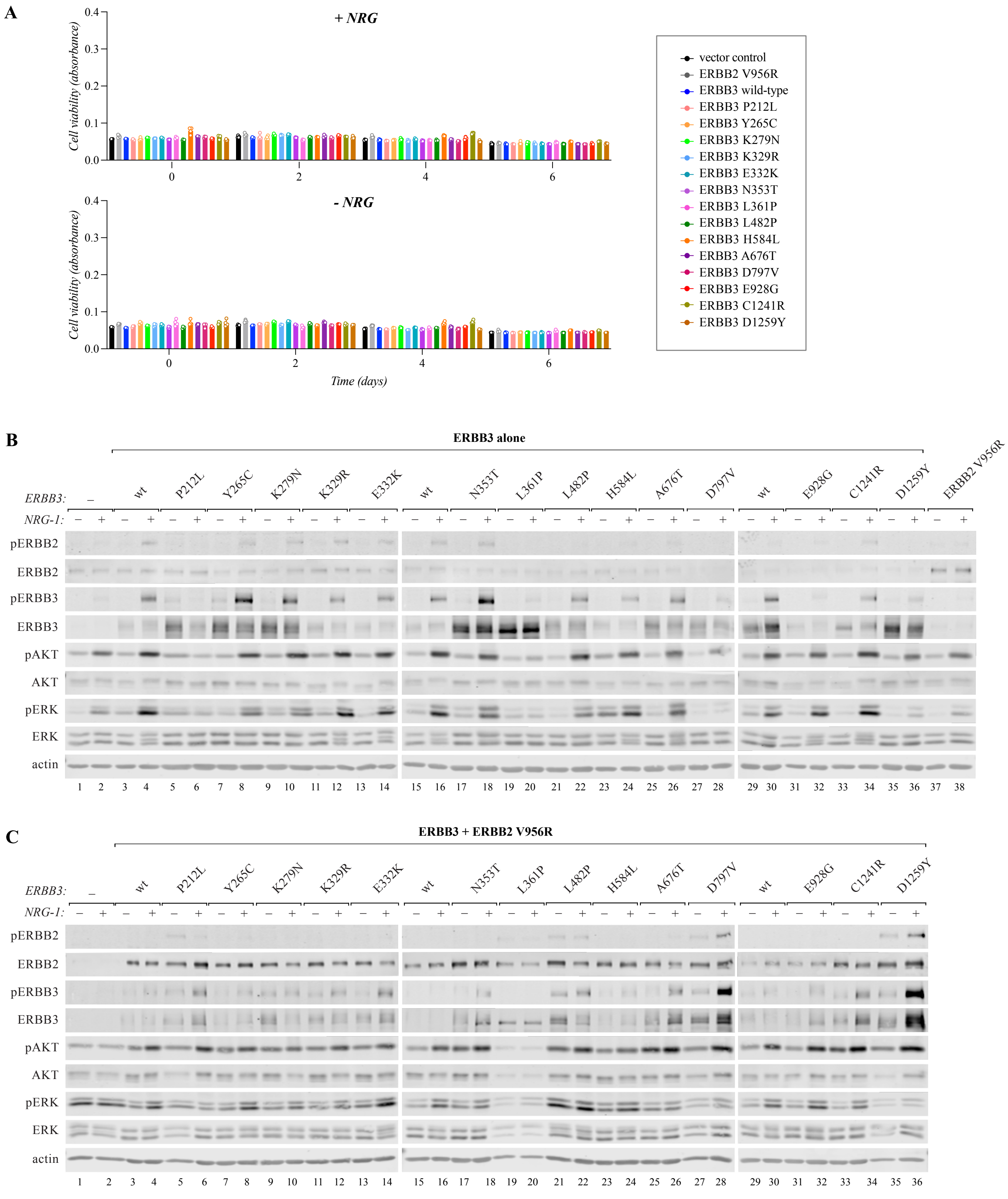

Supplementary Figure 8. ERBB3 variants in MCF-10A cells.

A: MCF-10A cells expressing indicated ERBB3 variants (without ERBB2 V956R co-expression) or ERBB2 V956R were cultured in the absence of serum and in the presence or absence of 50 ng/ml NRG-1 for 0, 2, 4, or 6 days. Cell viability was assessed from quadruplicate samples with the MTT assay. Mean and SD from an experiment representing one of three independent analyses is shown.

B-C: Western analysis of MCF-10A cells expressing the indicated ERBB3 variants in the absence (B) or presence (C) of the activator-incompetent ERBB2 V956R. For analysis shown in B, cells were starved in 0% serum overnight, and stimulated or not for 10 minutes with 50 ng/ml NRG-1. For analysis shown in C, cells were cultured either in 10% serum (– NRG), or starved in 0% serum and stimulated with 50 ng/ml of NRG-1 (+ NRG) for four days.

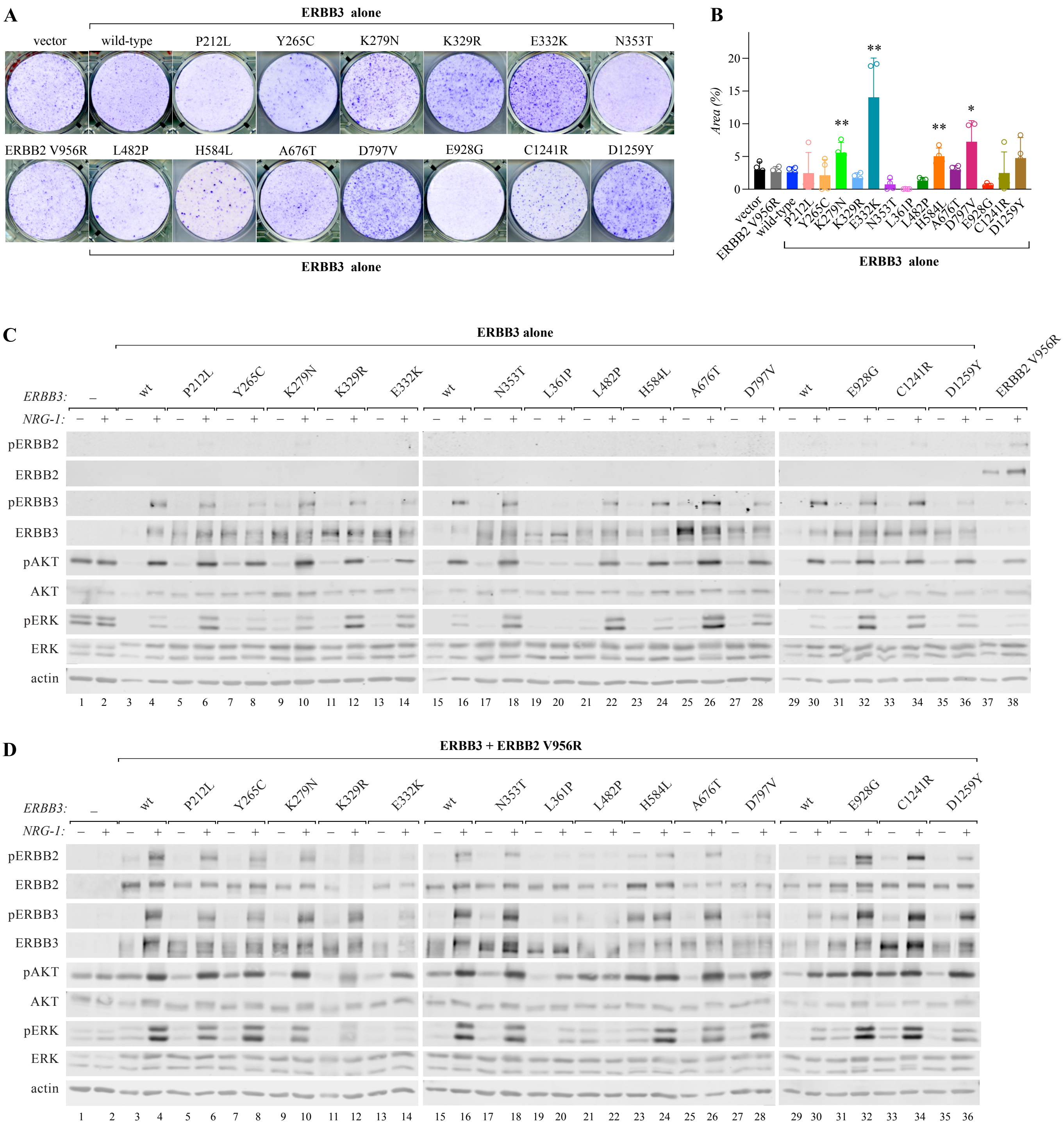

Supplementary Figure 9. ERBB3 variants in NIH 3T3 cells.

A: Focus formation analysis of NIH 3T3 cells expressing the indicated ERBB3 variants (without ERBB2 V956R co-expression) or ERBB2 V956R were cultured on 6-well plates for 2 weeks and stained with crystal violet.

B: Quantification of the focus formation data, such as shown in A. The area covered by foci was analyzed with ImageJ plugin ColonyArea. Mean and SD from four independent experiments are shown. *, P < 0.05; **, P < 0.01; unpaired two-sample t-test.

C-D: Western analysis of NIH 3T3 cells expressing the indicated ERBB3 variants in the absence (C) or presence (D) of the activator-incompetent ERBB2 V956R. Cells were starved overnight in 0% serum and stimulated with 50 ng/ml NRG-1 for 10 minutes or not.

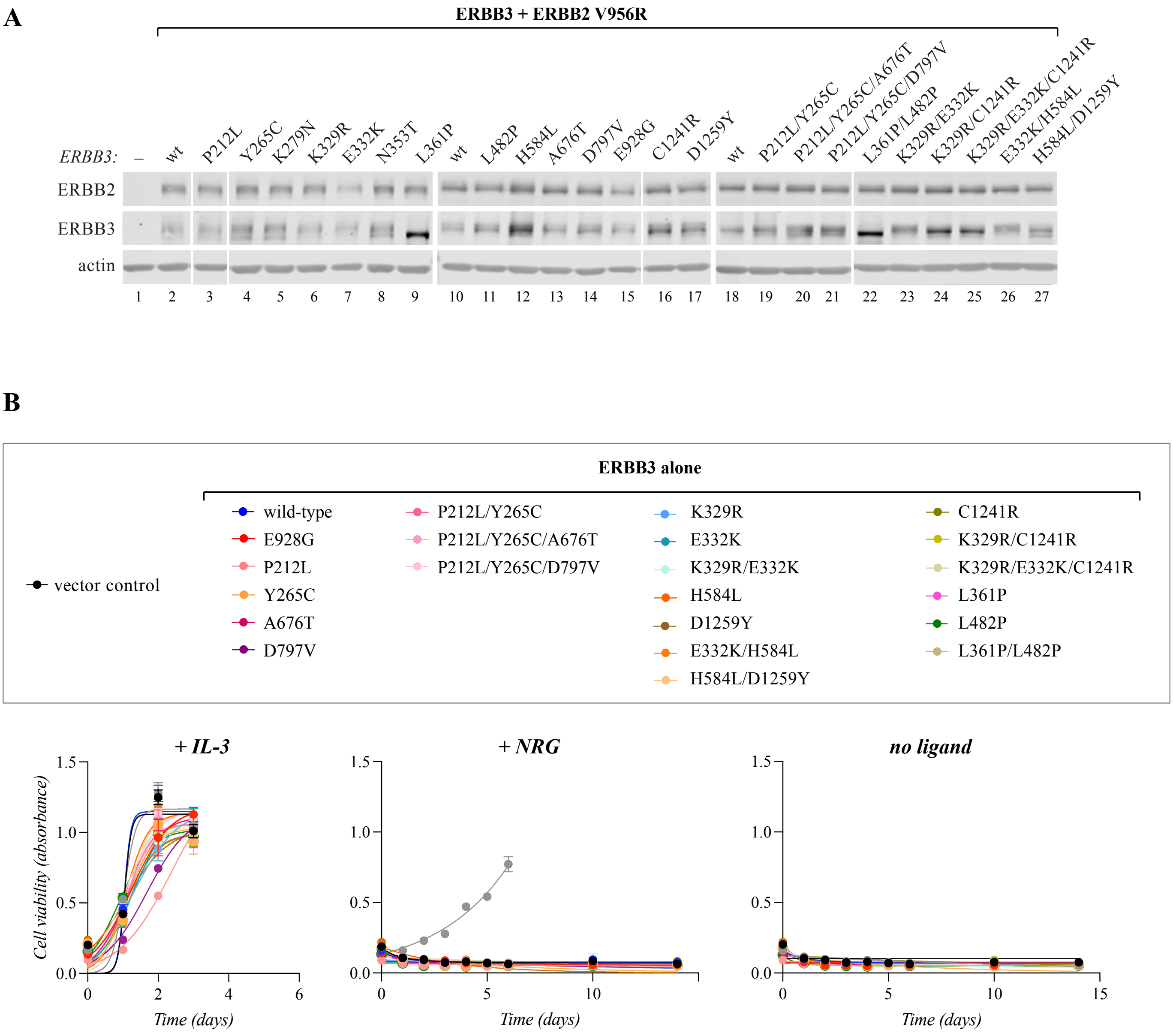

Supplementary Figure 10. Multiple ERBB3 mutations in Ba/F3 cells.

A: Western analysis of Ba/F3 cells expressing the indicated ERBB3 variants together with the activator-incompetent ERBB2 V956R. Cells were cultured in the presence of IL-3. Cell lysates were simultaneously analyzed in 3 different gels. Irrelevant lanes were excised.

B: Ba/F3 cells expressing the indicated ERBB3 variants (without ERBB2 V956R co-expression) or ERBB2 V956R were cultured in the presence of IL-3 or 20 ng/ml NRG-1, or in the absence of both (no ligand). Cell viability was assessed from quadruplicate samples with MTT assay. Mean and SD from an experiment representing one of four independent analyses is shown.

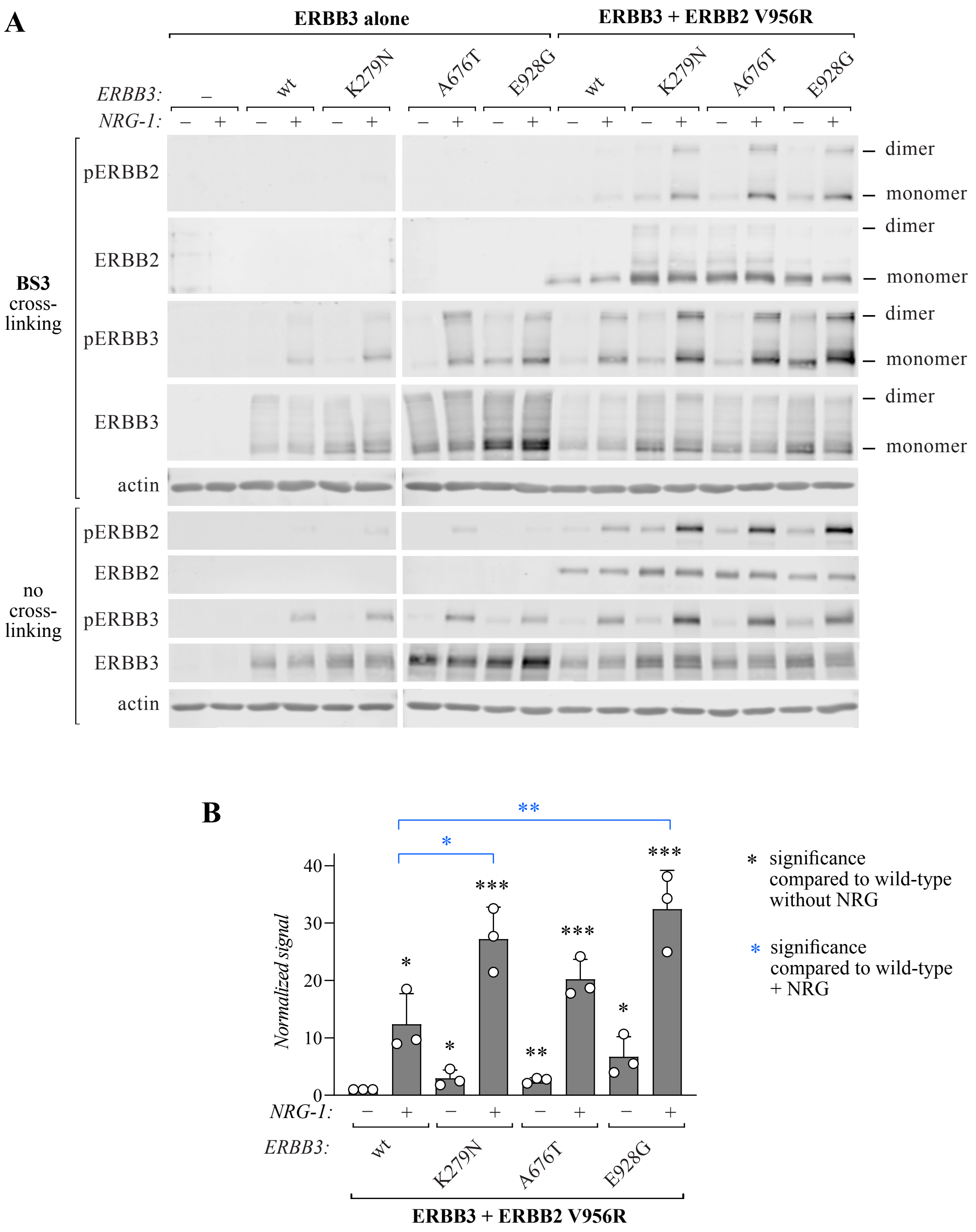

Supplementary Figure 11. Dimerization of ERBB3 variants.

A: NIH 3T3 cells stably expressing wild-type or mutant ERBB3 alone or together with activator-incompetent ERBB2 V956R were stimulated for 10 minutes with 0 or 10 ng/ml of NRG-1, crosslinked with membrane impermeable BS3 or not, and analyzed for ERBB dimerization by western blotting.

B: Densitometric quantification of phosphorylated ERBB3 dimers from western blot analyses such as shown in A. Mean and SD from three independent experiments are shown as normalized signal (to wild-type cells without NRG-1 stimulation). *, $P < 0.05$; **, $P < 0.01$; ***, $P < 0.001$; unpaired two-sample t test.

A

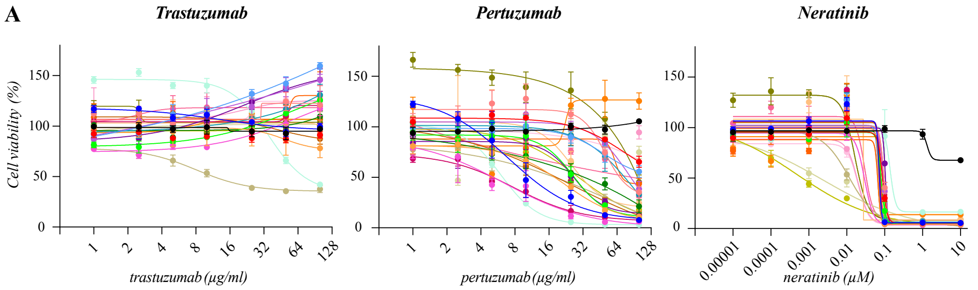

+NRG-1

Variant	Trastuzumab $IC_{50} \pm SD$ (µg/ml)	P (vs. wt)	n	Pertuzumab $IC_{50} \pm SD$ (µg/ml)	P (vs. wt)	n	Neratinib $IC_{50} \pm SD$ (nM)	P (vs. wt)	n
wild-type	58.5 ± 36.7		3	39.3 ± 20.1		3	89.3 ± 23.1		3
E928G	42.9 ± 41.8	0.39	3	48.8 ± 27.8	0.66	3	72.9 ± 10.6	0.33	3
P212L	46.8 ± 38.5	0.43	3	48.5 ± 14.5	0.56	3	66.4 ± 1.6	0.16	3
Y265C	65.8 ± 24.2	0.72	3	37.5 ± 14.3	0.90	3	91.4 ± 28.3	0.92	3
K279N	67.8 ± 24.1	0.77	3	16.8 ± 7.2	0.14	3	69.3 ± 0.9	0.21	3
K329R	45.0 ± 39.4	0.41	3	77.9 ± 20.7	0.08	3	102 ± 42.3	0.67	3
E332K	88.1 ± 59.5	0.82	3	67.7 ± 44.5	0.37	3	101 ± 23.1	0.57	3
N353T	105 ± 7.4	0.36	3	60.7 ± 21.7	0.28	3	70.4 ± 9.5	0.26	3
L361P	40.8 ± 22.3	0.29	3	9.1 ± 7.0	0.07	3	66.3 ± 5.9	0.17	3
L482P	28.1 ± 17.5	0.16	3	52.9 ± 14.3	0.39	3	277 ± 292	0.33	3
A676T	55.5 ± 46.5	0.59	3	18.2 ± 6.7	0.16	3	80.1 ± 17.8	0.61	3
D797V	69.5 ± 21.7	0.80	3	32.1 ± 20.9	0.69	3	149 ± 67.2	0.22	3
D1259Y	50.6 ± 37.3	0.48	3	41.9 ± 16.8	0.87	3	141 ± 47.5	0.17	3
K329R+E332K	30.2 ± 29.1	0.21	3	58.3 ± 9.3	0.21	3	311 ± 229	0.17	3
L361P+L482P	28.2 ± 24.6	0.18	3	14.3 ± 7.0	0.11	3	29.6 ± 24.4	0.04	3
H584L	86.9 ± 58.1	0.84	3	20.7 ± 22.1	0.34	3	125.1 ± 85.9	0.52	3
E332K+H584L	49.5 ± 35.8	0.45	3	7.9 ± 2.5	0.06	3	89.7 ± 69.9	0.99	3
H584L+D1259Y	56.0 ± 53.5	0.63	3	107.5 ± 22.0	0.02	3	17.3 ± 15.0	0.01	3
C1241R	72.4 ± 39.0	0.89	3	40.7 ± 42.3	0.96	3	73.2 ± 84.5	0.77	3
K329R+C1241R	51.5 ± 34.3	0.48	3	47.2 ± 37.5	0.76	3	78.8 ± 61.6	0.80	3
K329R+E332K+C1241R	70.6 ± 41.5	0.86	3	51.7 ± 39.1	0.65	3	536.7 ± 749.8	0.36	3
P212L+Y265C	83.3 ± 23.7	0.86	3	56.1 ± 33.5	0.50	3	248.9 ± 323.7	0.44	3
P212L+Y265C+A676T	49.9 ± 38.9	0.47	3	35.9 ± 12.9	0.82	3	5 734 ± 4201	0.08	3
P212L+Y265C+D797V	65.6 ± 24.3	0.72	3	62.1 ± 27.5	0.31	3	3 346 ± 4 705	0.30	3

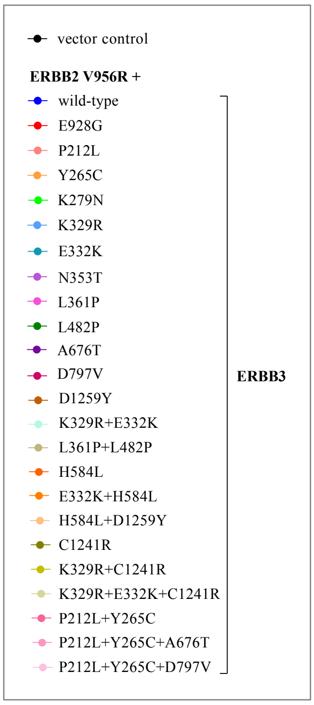

B

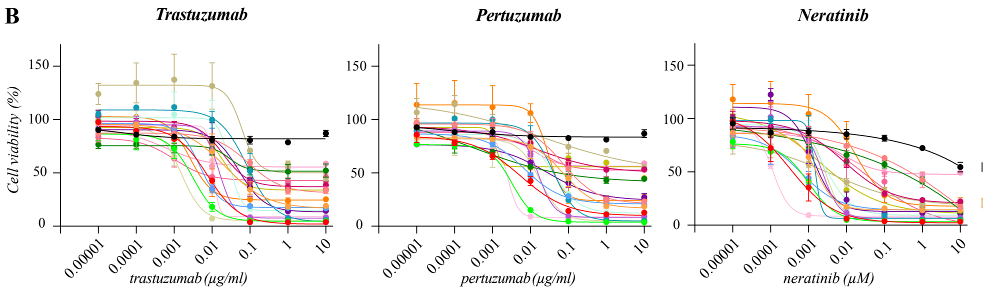

– NRG-1

Variant	Trastuzumab $IC_{50} \pm SD$ (ng/ml)	P (vs. wt)	n	Pertuzumab $IC_{50} \pm SD$ (ng/ml)	P (vs. wt)	n	Neratinib $IC_{50} \pm SD$ (nM)	P (vs. wt)	n
wild-type	77 400 ± 46 300		3	39 300 ± 20 100		3	89.3 ± 23.1		3
E928G	6.3 ± 3.5	0.04	3	9.6 ± 7.3	0.03	3	1.1 ± 0.6	0.003	3
P212L	407 ± 398	0.05	3	74.0 ± 32.0	0.03	3	297 ± 136	0.06	3
Y265C	26.0 ± 7.1	0.04	3	36.8 ± 24.8	0.03	3	3.0 ± 1.7	0.003	3
K279N	12.7 ± 7.2	0.04	3	7.3 ± 7.9	0.03	3	1.1 ± 0.4	0.003	3
K329R	80.1 ± 58.9	0.04	3	65.6 ± 34.5	0.03	3	8.1 ± 5.6	0.004	3
E332K	24.1 ± 13.0	0.04	3	33.8 ± 5.4	0.03	3	2.5 ± 0.9	0.003	3
N353T	150 ± 89.8	0.04	3	3 350 ± 4 700	0.04	3	6.6 ± 1.3	0.004	3
L482P	83.2 ± 82.1	0.04	3	28.9 ± 17.4	0.03	3	668 ± 421	0.08	3
A676T	48.2 ± 20.2	0.04	3	40.5 ± 35.3	0.03	3	4.3 ± 2.3	0.003	3
D797V	45.2 ± 12.6	0.04	3	29.0 ± 22.4	0.03	3	15.7 ± 6.4	0.006	3
K329R+E332K	42.4 ± 11.0	0.04	3	61.3 ± 20.4	0.03	3	5.1 ± 1.4	0.003	3
L361P+L482P	31.2 ± 21.6	0.04	3	3 340 ± 4 710	0.04	3	812 ± 942	0.25	3
E332K+H584L	3.4 ± 1.0	0.04	3	29.0 ± 6.3	0.03	3	24.3 ± 17.0	0.02	3
K329R+C1241R	18.3 ± 10.4	0.04	3	1.2 ± 0.04	0.03	3	1.6 ± 0.8	0.003	3
K329R+E332K+C1241R	3.2 ± 2.0	0.04	3	18.7 ± 2.9	0.03	3	4.2 ± 3.0	0.003	3
P212L+Y265C	4.1 ± 4.1	0.04	3	41.9 ± 39.3	0.03	3	3 386 ± 4 677	0.29	3
P212L+Y265C+A676T	1.0 ± 0.9	0.04	3	14.8 ± 11.9	0.03	3	14.6 ± 14.3	0.009	3
P212L+Y265C+D797V	4.9 ± 4.3	0.04	3	1.8 ± 0.5	0.03	3	0.05 ± 0.05	0.003	3

Supplementary Figure 12. Sensitivity of the ERBB3 variants to ERBB inhibitors.

Ba/F3 cells expressing the indicated ERBB3 variants in the presence of the activator-incompetent ERBB2 V956R were cultured in the presence (A) or absence (B) of 20 ng/ml NRG-1 and treated with trastuzumab, pertuzumab, or neratinib for 72 hours. Cells transduced with an empty vector and cultured in the presence of IL-3 served as a control for off-target toxicity. Cell viability was assessed from triplicate samples with MTT assay. IC50 values were calculated from three independent analyses. In the absence of NRG-1, all treatments resulted in significantly ($P < 0.05$ with unpaired two-sample t test) smaller IC50's when compared to cells expressing wild-type ERBB3 and stimulated with NRG-1, with the exceptions indicated with red *P* values in panel B. In the presence of NRG-1, all cell lines were resistant to all drugs ($P \geq 0.05$ for IC50 compared to wild-type ERBB3 + NRG-1) with the exception indicated with blue *P* value in panel A. No lines surviving the absence of NRG-1 and IL-3 could be established from cell populations expressing wild-type ERBB3, or the L361P or D1259Y mutation.

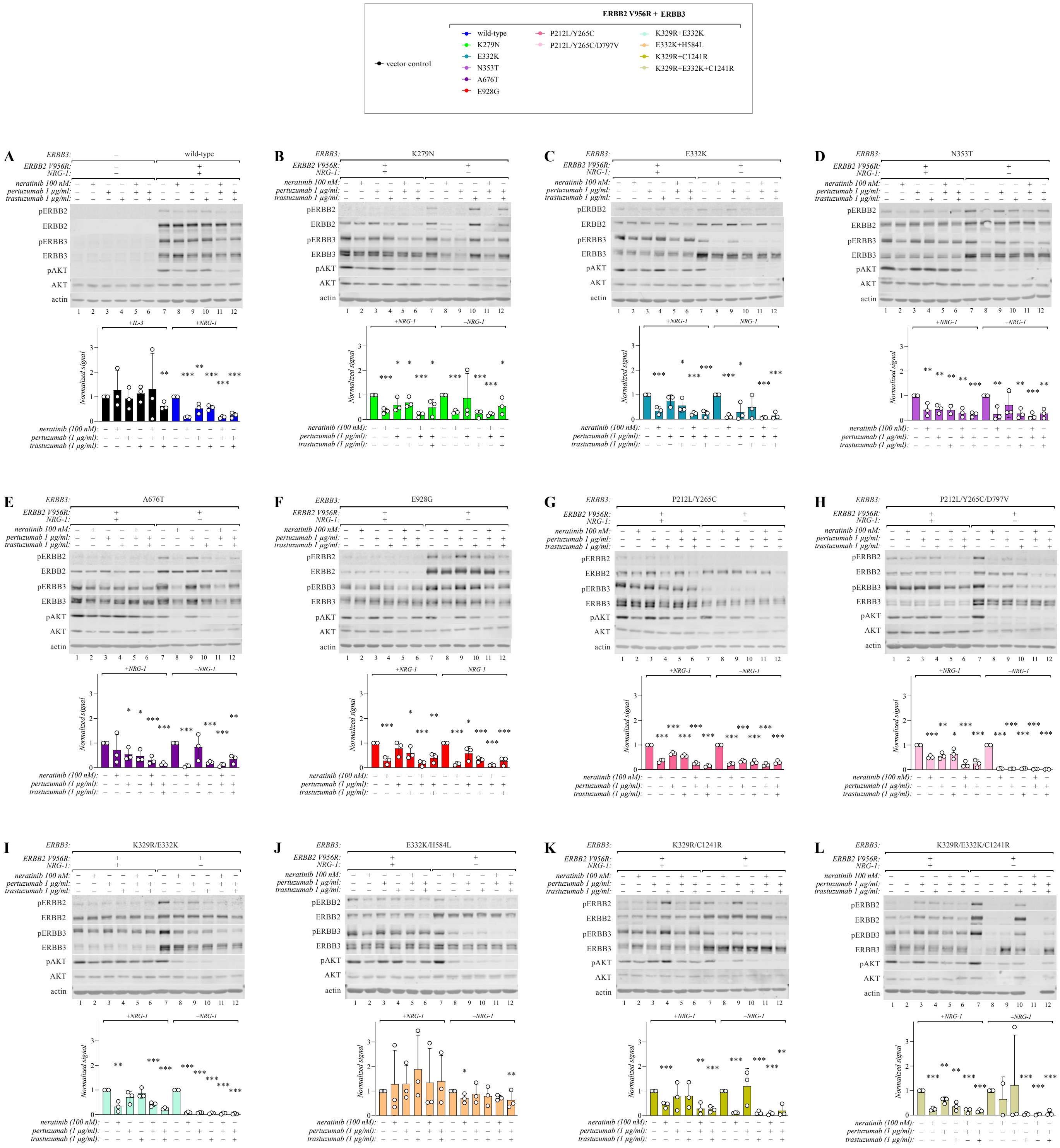

Supplementary Figure 13. Effects of ERBB-targeting compounds on ERBB signaling.
Ba/F3 cells expressing the indicated ERBB3 variants together with the activator-incompetent ERBB2 V956R were maintained without IL-3 in the presence or absence of 20 ng/ml NRG-1. To carry out drug sensitivity assays, buffer control, 100 nM neratinib, 1 µg/ml pertuzumab, and/or 1 µg/ml trastuzumab were added to the culture medium for five hours. Cells were lysed and subjected to western analysis with the indicated antibodies. Cells transduced with an empty vector and cultured in the presence of IL-3 served as a control (panel A, lanes 1-6). Densitometric quantification of AKT phosphorylation status from western blot analyses is shown below each blot. The mean and SD are shown from three independent experiments. *, $P < 0.05$: **, $P < 0.01$; ***, $P < 0.001$; unpaired two-sample t test; comparison to cells not treated with drugs.
